# Injectable pH Responsive Conductive Hydrogel for Intelligent Delivery of Metformin and Exosomes to Enhance Cardiac Repair after Myocardial Ischemia‐Reperfusion Injury

**DOI:** 10.1002/advs.202410590

**Published:** 2025-02-18

**Authors:** Nianlan Cheng, Qiao Luo, Yongqing Yang, Ni Shao, Tianqi Nie, Xiujiao Deng, Jifeng Chen, Siqi Zhang, Yanyu Huang, Kuan Hu, Liangping Luo, Zeyu Xiao

**Affiliations:** ^1^ The Guangzhou Key Laboratory of Molecular and Functional Imaging for Clinical Translation Department of Radiology and Nuclear Medicine The First Affiliated Hospital of Jinan University Guangzhou 510630 China; ^2^ Central laboratory Guangzhou Twelfth People's Hospital Guangzhou 510620 China; ^3^ State Key Laboratory of Bioactive Substance and Function of Natural Medicines Institute of Materia Medica Chinese Academy of Medical Sciences & Peking Union Medical College Beijing 100050 China; ^4^ Department of Biochemistry and Molecular Medicine University of California Davis Sacramento CA 95817 USA; ^5^ Department of Radiology and Nuclear Medicine The Fifth Affiliated Hospital of Jinan University (Shenhe People's Hospital) Heyuan 517000 China

**Keywords:** cardiac repair, exosomes, injectable conductive hydrogel, metformin, myocardial ischemia‐reperfusion injury

## Abstract

Myocardial ischemia‐reperfusion injury (MIRI) is a leading cause of complications and high mortality associated with acute myocardial infarction. Injectable hydrogel emerges as a promising biomaterial for myocardial repair due to their ability to mimic the mechanical and electrophysiological properties of heart tissue. In this study, an injectable conductive hydrogel is developed that responds to the weakly acidic microenvironment of ischemic injury, enabling the intelligent release of metformin and exosomes to enhance cardiac repair following MIRI. This multifunctional hydrogel demonstrates self‐healing properties, shear‐thinning injectability, electrical conductivity, and an elastic modulus comparable to natural myocardium, alongside excellent biocompatibility. At the cellular level, the hydrogel system exhibits significant antioxidant, anti‐apoptotic, improvement of electrophysiological characteristics, mitochondrial protection and angiogenic effects, with transcriptome sequencing revealing the effective activation of the PI3K/AKT, VEGF, and AMPK signaling pathways. In vivo studies further confirm that the hydrogel treatment reduces infarct size, cardiac fibrosis and incidence of arrhythmia, while improving ventricular ejection fraction and facilitating the restoration of cardiac function after MIRI. In conclusion, an injectable pH‐responsive conductive hydrogel is presented that enables the intelligent delivery of metformin and exosomes, offering a promising and novel therapeutic approach for enhancing cardiac repair and treating MIRI.

## Graphical abstract

Injectable pH responsive conductive hydrogel for intelligent delivery of metformin and exosomes to alleviate myocardial ischemia‐reperfusion injury. The hydrogel responds to the weakly acidic microenvironment of ischemic injury and can significantly reduce the production of intracellular ROS and enhance cardiac conduction, thereby resisting apoptosis, and to promote angiogenesis, thus effectively promoting myocardial repair and improving cardiac function.

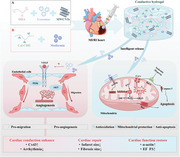

## 1 Introduction

Coronary artery disease (CAD) is one of the major causes of heart failure, a common ischemic heart disease in clinical practice, and the mortality rate is increasing year by year.^[^ ^1^, ^2^ ^]^ Timely restoration of ischemic myocardial blood flow by percutaneous coronary intervention (PCI) is an effective means to save dying cardiomyocytes, minimize the extent of myocardial infarction (MI), and reduce mortality.^[^ ^3^, ^4^ ^]^ However, restoration of blood flow in the reperfused area may induce secondary damage to cardiomyocytes, which severely impairs cardiac function, even aggravates the extent of MI, and ultimately leads to ventricular remodeling. Myocardial ischemia‐reperfusion injury (MIRI) involves a variety of pathophysiological processes such as apoptosis, mitochondrial damage, oxidative stress, and endothelial cell dysfunction,^[^ ^5^, ^6^ ^]^ and it is important to explore comprehensive therapeutic strategies that can effectively attenuate apoptosis, oxidative stress, and stimulate ischemic angiogenesis to restore circulation for myocardial tissue repair after injury.

Metformin is a commonly used diabetes drug, but its efficacy is not limited to glycemic control. Several studies have now shown that metformin helps to reduce MIRI and delay ventricular remodeling.^[^ ^7^ ^]^ Although the mechanism by which the drug acts on cardioprotective effects is not yet fully understood, the widely accepted mechanism of action is activation of the AMP‐activated protein kinase (AMPK) pathway.^[^ ^8^ ^]^ Metformin activates the AMPK pathway by blocking electron transport in the mitochondrial respiratory chain complex I, decreasing adenosine triphosphate (ATP) synthesis, and increasing intracellular AMP levels and the ratio of AMP/ATP.^[^ ^9^, ^10^ ^]^ AMPK serves as an energy sensor in the myocardial energy metabolism AMPK acts as an energy sensor in myocardial energy metabolism, activating the ATP‐generating pathway during energy depletion and reducing energy expenditure to provide myocardial protection.^[^ ^10^, ^11^ ^]^ In addition, AMPK has many downstream targets, such as antioxidant enzymes and mitochondrial biogenesis transcription factors, which help maintain cardiomyocyte viability during myocardial ischemia.^[^ ^12^ ^]^ Nevertheless, metformin has certain limitations in the treatment of MIRI. Its efficacy is primarily manifested in the enhancement of myocardial energy metabolism and the reduction of oxidative stress. Further improvements in efficacy are necessary. The combination of metformin with treatment methods that promote cardiac repair may offer a more promising treatment strategy.

In recent years, the development of stem cell regenerative medicine has brought new hope for the treatment of cardiovascular diseases. Several studies have demonstrated that stem cells can promote the repair of damaged myocardial tissues through paracrine exosomes and cytokines.^[^ ^13^, ^14^ ^]^ Exosomes are the key secretion product of stem cells and the main contributing component in providing tissue repair efficacy. Exosomes have a nanoscale size and immune tolerance comparable to that of stem cells, and miRNAs transported by exosomes play an important role in the exchange of cellular materials and information by regulating and facilitating intercellular communication through the reception of epigenetic information from cells.^[^ ^15^, ^16^ ^]^ In recent years, several studies have demonstrated that mesenchymal stem cell exosomes (MSC‐Exos) are involved in myocardial tissue repair after myocardial damage through multiple pathways, especially in promoting angiogenesis.^[^ ^17^, ^18^ ^]^ Although current literature does not explicitly define a direct synergistic relationship between metformin and exosomes, our observations suggest the existence of a potential mechanistic interplay between the two. Metformin exerts anti‐apoptotic and antioxidant effects,^[^ ^19^ ^]^ which can improve the local microenvironment and restore mitochondrial function in ischemic tissues.^[^ ^7^ ^]^ These effects may enhance the uptake and biological activity of exosomes, thereby promoting exosome‐mediated tissue repair and accelerating myocardial regeneration and functional recovery. Moreover, metformin and exosomes may synergistically activate endothelial cell function and stimulate angiogenesis through multiple pathways, further supporting tissue repair.^[^ ^13^, ^20^ ^]^ This potential interaction underscores the therapeutic promise of combining metformin and exosomes for the treatment of MIRI.

In the MIRI environment, local tissue inflammation and oxidative stress are significantly elevated. The sustained activation of AMPK at the site of myocardial injury can be achieved through the localized, high‐concentration release of metformin, which more effectively suppresses inflammation and oxidative stress while protecting endothelial function. In contrast, oral administration often results in insufficient local drug concentrations due to systemic distribution. For exosome‐based therapy, direct injection is the most common delivery method. However, rapid clearance and short in vivo half‐lives of exosomes significantly limit their therapeutic efficacy, particularly in myocardial tissue regeneration and repair, which requires sustained delivery and repeated dosing.^[^ ^21^ ^]^ Using biomaterials as a carrier for the dual delivery of metformin and exosomes offers a promising solution to address these challenges and enhance therapeutic outcomes.

Hydrogel is a 3D porous polymer network with good hydrophilicity and biocompatibility. Implantation of injectable hydrogel into the damaged region can provide mechanical support to the myocardial wall, reducing the mechanical load on the heart and the pressure on the left ventricular wall, and preventing deterioration of the mechanical environment, thereby preventing ventricular remodeling.^[^ ^22^, ^23^ ^]^ Based on these observations, hydrogel with conductive properties can further optimize the conductive microenvironment in the damaged region, acting as a bridge between healthy tissue and the damaged region, allowing electrical impulses to propagate through the region to enhance synchronized myocardial contraction, promoting cardiomyocyte maturation and restoration of cardiac function.^[^ ^24^ ^]^ Therefore, the development of conductive hydrogel with mechanical properties matching those of natural myocardium would be a promising solution for the treatment of MIRI. More importantly, the hydrogel can serve as an optimal vehicle for the delivery of drugs and bioactive factors, ensuring efficient and sustained release of therapeutic agents, facilitating myocardial regeneration and repair, and promoting the recovery of cardiac function throughout the entire cycle. Based on this, a novel injectable conductive hydrogel loaded with metformin/MCS‐Exos was designed to establish the dynamic cross‐linking of oxidized hyaluronic acid (OHA) and carbohydrazide‐modified collagen (Col‐CDH) via Schiff base reaction in this research, the Schiff base bond is stable at pH 7.4 but is disrupted in an acidic environment (pH 6.8),^[^ ^25^ ^]^ enabling an on‐demand release of metformin and exosomes in myocardial weakly acidic ischemic infarction microenvironment. At the same time, carboxylated multi‐walled carbon nanotubes (MWCNT) were introduced as a conductive material to form a stable three‐dimensional conductive network together with the above system.

**Scheme 1:**
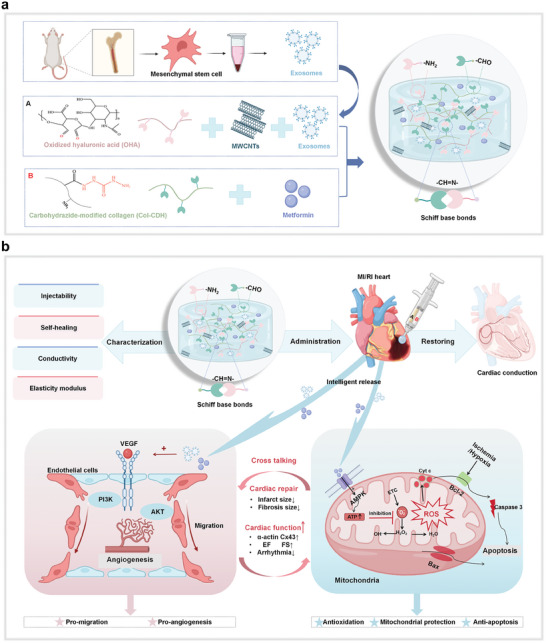
Scheme design of the injectable pH‐responsive multifunctional conductive hydrogel. a) Schematic diagram of exosome extraction and hydrogel synthesis. Exosomes are extracted from rat bone marrow mesenchymal stem cells and the hydrogel system is constructed by Schiff base reaction based on OHA and Col‐CDH. b) Hydrogel characterization and therapeutic mechanism. The hydrogel responds to the weakly acidic microenvironment of ischemic infarcts to achieve Intelligently release therapeutic factors on demand. The hydrogel can significantly enhance cardiac conduction and reduce the production of intracellular reactive oxygen species, thereby resisting apoptosis, and to promote angiogenesis, thus effectively promoting myocardial repair and improving cardiac function.

The primary objectives of this study were to develop a conductive, injectable hydrogel therapeutic system designed for the efficient and intelligent delivery of a novel combination of metformin and exosomes, to investigate the synergistic effects of metformin and exosomes, and to demonstrate the therapeutic potential of this innovative approach. This combination aims not only to address the rapid clearance of exosomes but also to provide a localized and sustained release of metformin, thereby maximizing the synergistic effects of these two agents for myocardial repair and functional recovery. To achieve this, we designed a hydrogel system capable of intelligently releasing therapeutic agents in response to the mildly acidic microenvironment (pH 6.8) characteristic of myocardial ischemic regions during MIRI. This study systematically evaluated the efficacy of the dual‐loaded hydrogel for treating MIRI and promoting cardiac repair through a series of in vitro and in vivo experiments. Additionally, transcriptome RNA sequencing was employed to investigate the underlying mechanisms of hydrogel‐based therapy. Specifically, we examined the roles of hydrogel system in anti‐apoptotic and anti‐oxidative stress pathways, mitochondrial protection, electrophysiological stabilization, and angiogenesis promotion at cellular and bioinformatics levels. Finally, the in vivo therapeutic efficiency of the hydrogel was assessed, focusing on its ability to improve the structural integrity and function of the damaged myocardium, enhance myocardial signal transduction, and support cardiac repair (**Scheme** **1**).

## 2 Results and Discussion

### 2.1 Preparation and Characterization of Hydrogel

**Figure 1:**
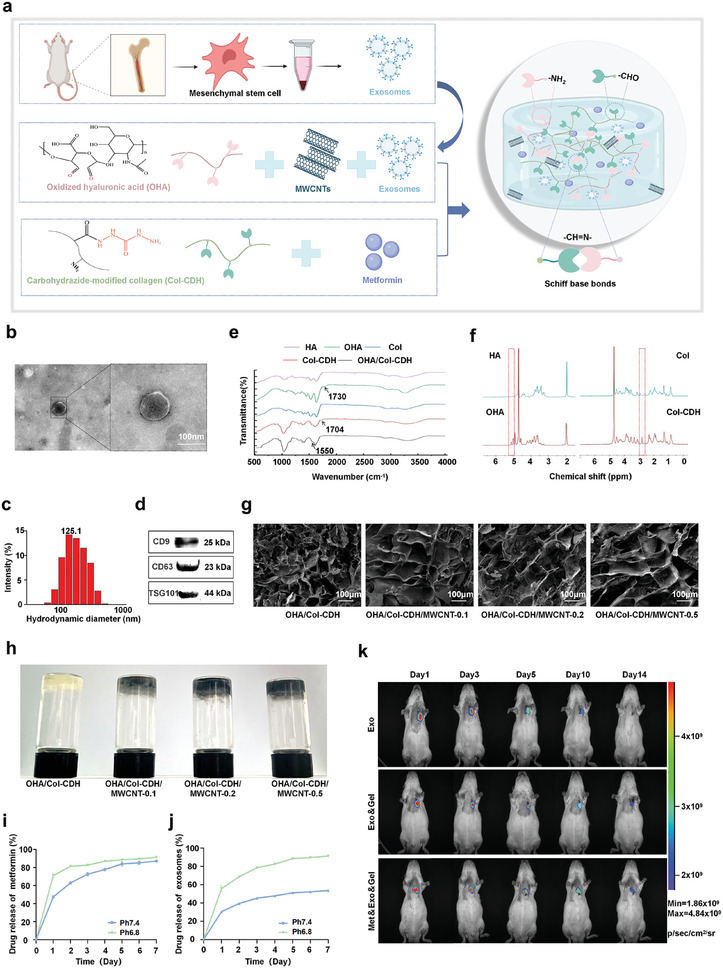
Exosome extraction and synthesis of hydrogels. a) Schematic diagram of bone marrow mesenchymal stem cell exosome extraction and hydrogel system synthesis. b) TEM images of MSC‐Exos. Scale bars: 100 nm. c) Particle size diagram of MSC‐Exos. d) Western blot image of CD9, CD63 and TSG101 protein expression in exosomes. e) FTIR spectra of HA, OHA, Col, Col‐CDH, and OHA/Col‐CDH. f) ^1^H NMR spectra of HA, OHA, Col, and Col‐CDH. g) SEM images of hydrogels. Scale bars: 100 µm. h) The “vial inversion method” shows the hydrogel synthesis diagram. i) Curve of metformin release from hydrogels. j) Curve of exosomes release from hydrogels. k) The fate of exosomes in vivo was tracked by bioluminescence imaging. Statistical data by means ± SD (n = 3).

Bone marrow mesenchymal stem cells (MSCs) were harvested according to the procedures described in our previous research.^[^ ^26^ ^]^ Exosomes were extracted from MSCs using a kit and subsequently characterized. Based on it, a versatile hydrogel was synthesized by cross‐linking Col‐CDH with OHA and incorporating metformin, MSC‐Exos and MWCNT (**Figure** **1**a). The exosomes exhibited a characteristic double‐layered membrane structure (Figure 1b) in transmission electron microscopy (TEM) with a maximum distribution intensity at the 125.1 nm (Figure 1c). Western blot analysis confirmed that the isolated exosomes expressed the specific surface markers CD9, CD63, and TSG101 (Figure 1d). It has been shown that the internalization of exosomes by target cells involves endocytosis, facilitating the delivery of lipids, proteins, mRNAs, or miRNAs. Accordingly, after co‐incubation of exosomes with H9c2 cells for 24 hours, fluorescence microscopy revealed that PKH26‐labelled exosomes localized around the nucleus of H9c2 and human umbilical vein endothelial cell (HUVEC) (Figure S1, Supporting Information), indicating successful endocytosis of the therapeutic cargoes into the cytoplasm. The collective evidence suggests that the exosomes were accurately isolated from the MSC‐conditioned medium, resuspended, and purified in accordance with the established criteria for exosome identification.

Oxidation of the proximal hydroxyls in the sugar rings of hyaluronic acid (HA) was achieved by the synthesis of OHA with dialdehyde groups, which was carried out using sodium periodate. The peak at 1730 cm^−1^ (C═O) observed in the Fourier transform infrared spectroscopy (FTIR) spectrum (Figure 1e) confirmed the successful oxidation of HA, which was consistent with the findings reported in the literature. Col‐CDH was obtained through the coupling of CDH with acylhydrazine bonds onto collagen chains using the NHS/EDC method. The FTIR spectrum (Figure 1e) also confirmed the successful hydrazide modification on collagen through the appearance of the stretching vibration peak at 1704 cm^−1^. In the FTIR spectra of the lyophilised hydrogels, a peak at 1550 cm^−1^ was observed, confirming the formation of C═N bonds between aldehyde groups on OHA and hydrazide groups on Col‐CDH. The ^1^H NMR spectra of OHA and Col‐CDH were presented in Figure 1f. The successful oxidation of hyaluronic acid, CDH modification of collagen, and hydrogel synthesis were confirmed by forenamed results.

Scanning electron microscopy (SEM) revealed that the hydrogel had a porous and irregular network structure (Figure 1g). OHA/Col‐CDH had the smallest pore size (about 40 µm) and the pore sizes of other hydrogels were basically the same (about 100 µm). It may be that the carboxyl group in MWCNT conjugated to Col‐CDH consumed a certain amount of amino group, which led to a reduction in crosslink density between the amido groups of Col‐CDH and the aldehyde groups of OHA. The gelation time of the hydrogel was evaluated using the inverted bottle method, which showed that the gelation time decreased with higher concentrations of Col‐CDH and OHA (Figure 1h). We chose a 10% concentration for both OHA and Col‐CDH, which gave a gelation time of approximately 20 seconds, ensuring rapid in situ gel formation and injectability (Figure S2, Supporting Information).

To investigate the drug release characteristics of hydrogel, the in vitro release of metformin and MSC‐Exos loaded hydrogels was measured at two different pH values: pH 7.4 (representing physiological pH) and pH 6.8 (representing the microenvironment of myocardial ischaemic injury).^[^ ^27^, ^28^ ^]^ The release profiles of metformin (Figure 1i) and exosomes (Figure 1j) showed an initial burst release at both pH values. The cumulative release of metformin from hydrogel was 91.48% ± 1.00% and 87.23% ± 0.56% at pH 6.8 and 7.4, respectively. The exosome‐release profile showed an initial burst followed by a sustained release phase, reaching equilibrium after 120 hours at both pH 6.8 and pH 7.4. The cumulative release rate of MSC‐Exos from the hydrogel was higher at pH 6.8. It can be seen that both metformin and exosomes are released rapidly in the microenvironment of myocardial injury because the imine bonds formed by the Schiff base reaction are cleaved in an acidic environment, whereas metformin shows a more pronounced early release compared to exosomes because metformin contains amino groups and the imine bonds formed in the hydrogel system are also cleaved in an acidic environment. This dynamic and reversible chemical bond allows for smart and pH‐responsive for metformin/exosomes release while maintaining its activity. The expeditious release of metformin and exosomes can engage in the pathological process of anti‐oxidation and anti‐apoptosis in the initial phase of myocardial injury. Meanwhile, the sustained release of exosomes can facilitate angiogenesis and assume a pivotal role in the subsequent phases of blood circulation reconstruction following myocardial injury which would be substantiated through subsequent experiments. Meanwhile, naked exosomes, as well as exosomes and exosomes combined with metformin embedded within OHA/Col‐CDH/MWCNT hydrogels, were administered to a rat model of MIRI. The distribution and retention of exosomes were evaluated through bioluminescence imaging to trace their in vivo fate. Fourteen days post‐injection, bioluminescence signals remained detectable in both the Exo&Gel and Met&Exo&Gel groups, whereas negligible fluorescence signals were observed in the naked Exo group (Figure 1k). These results highlight the ability of OHA/Col‐CDH/MWCNT hydrogels to significantly improve the retention, stability, and sustained release of MSC‐Exos.

### 2.2 Multifunctional Properties of the Hydrogel

**Figure 2:**
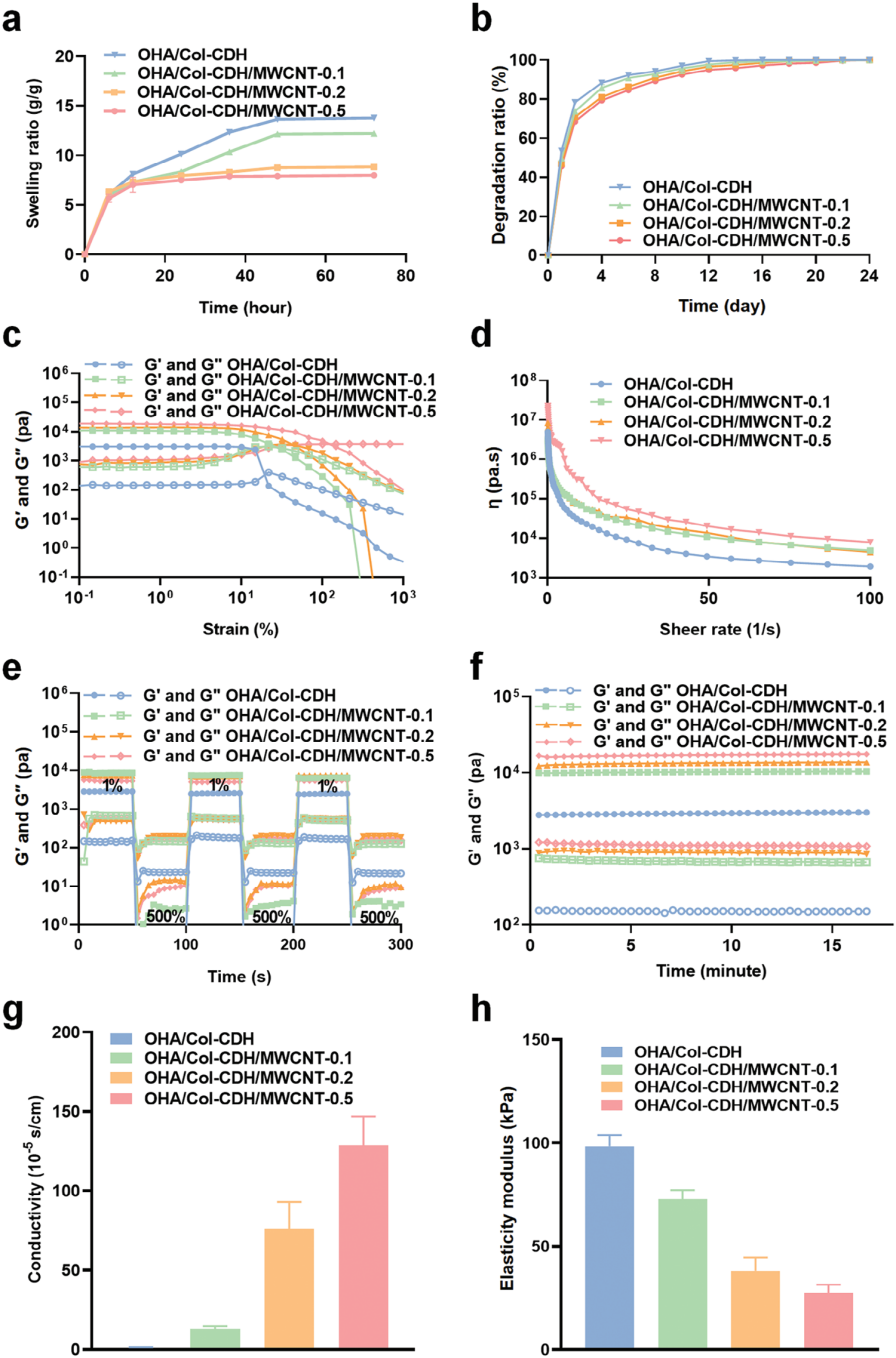
Characteristics of multifunctional hydrogels. a) Swelling curve of hydrogels. b) Degradation curve of hydrogels. c) The storage modulus (G′) and loss modulus (G″) of the hydrogels under varying oscillatory strains. d) The relationship between viscosity and shear rate in the hydrogels. e) The self‐healing ability of hydrogels when subjected to alternating strains. f) Rheological assessments conducted in time‐sweep mode for the hydrogels. g) The electrical conductivity of the hydrogels. h)The elasticity modulus of the hydrogels. Statistical data by means ± SD (n = 3).

The swelling behavior of the hydrogels was shown in **Figure** **2**a, which showed that all hydrogels reached swelling equilibrium within 48 h. The swelling rate decreased with increasing concentrations of MWCNT, in agreement with previous results.^[^ ^29^ ^]^ Among the samples, OHA/Col‐CDH/MWCNT‐0.5 had the lowest swelling ratio, while OHA/Col‐CDH had the highest one. The moderate swelling behavior and network structure of hydrogel offer potential advantages for use in confined in vivo environments. Degradation studies were performed in PBS solution (pH ≈ 7.4) with collagenase (0.2 U) to simulate biological degradation.^[^ ^30^ ^]^ As shown in Figure 2b, all hydrogels showed adequate degradation rates, achieving complete degradation in vivo after 21 days, ensuring that any remaining metformin and MSC‐Exos were released as the hydrogels degraded, meeting the requirements for myocardial biomaterials.

The rheological properties of the hydrogels were evaluated using a rotational rheometer. Strain scans (Figure 2c) demonstrated that all hydrogels remained stable under low strain. As the strain exceeded the critical value (G′ is lower than G″), the hydrogel network collapsed, resulting in the transformation of the solid hydrogel into a fluid state. As the shear rate increases, the hydrogel exhibits shear‐thinning properties (Figure 2d), which ensured the injectability of the multifunctional hydrogel. This change is attributed to the cleavage and separation of Schiff base bonds under high shear strain. The shear‐thinning characteristics and rapid network recovery of hydrogels facilitate their injection and prompt recovery.^[^ ^22^, ^31^ ^]^ In alternating strain scans (Figure 2e), all hydrogels maintained a stable network structure at low strain (1%). At high strain (500%), G′ < G″ indicates that the hydrogel crosslinking network collapses. Following a transition from high to low strain, both G′ and G″ rapidly returned to their initial values, indicating the restoration of the cross‐linked network within the hydrogel. This observation suggests that the hydrogel exhibits excellent self‐healing properties. The gelation kinetics were evaluated in the dynamic time‐scanning mode, and the results are shown in Figure 2f. It can be observed that the storage modulus (G′) values of all the hydrogels were greater than the loss modulus (G″) value, indicating that all hydrogels could rapidly form stable cross‐linked structures. In general, the characterization of hydrogel rheological parameters indicates that the hydrogel system can quickly form a stable cross‐linked structure, and has self‐healing and injectable properties, which is suitable for the comprehensive treatment of MIRI.

MWCNT exhibit excellent conductivity, and the conductivity of hydrogel is of paramount importance for the transmission of electrical signals in infarcted myocardium.^[^ ^32^ ^]^ First, the conductivity of the hydrogel was measured using a four‐point probe (Figure 2g). The conductivity of OHA/Col‐CDH hydrogels without conductive components MWCNT was found to be 1.97×10^−5^ S cm^−1^. Following the addition of MWCNT, the conductivities of the OHA/Col‐CDH/MWCNT‐0.1, OHA/Col‐CDH/MWCNT‐0.2 and OHA/Col‐CDH/MWCNT‐0.5 hydrogels were observed to increase to 1.29×10^−4^ S cm^−1^, 7.61×10^−4^ S cm^−1^ and 1.28×10^−3^ S cm^−1^, respectively. The conductivities of the OHA/Col‐CDH/MWCNT‐0.1 and OHA/Col‐CDH/MWCNT‐0.2 hydrogels were found to be consistent with the conductivity range of native cardiac tissue (≈10^−4^ S cm^−1^),^[^ ^33^, ^34^ ^]^ as reported in reference. Subsequently, electrochemical studies were conducted using an electrochemical workstation with a three‐electrode system. The cyclic voltammetry (CV) curve (Figure S3, Supporting Information) of the hydrogel exhibited a symmetrical hysteresis loop and a pair of redox peaks. The enclosed area of the CV curve is positively correlated with the capacitance of conductive materials.^[^ ^35^ ^]^ The addition of MWCNT resulted in an increase in the area of the hysteresis loop and the redox peak becoming more pronounced, indicating that the incorporation of MWCNT enhanced the conductivity of the hydrogels. The excellent electrical conductivity renders it an optimal candidate for the repair of heart tissue.

An ideal hydrogel for myocardial repair should have good mechanical properties to maintain its integrity during use. The mechanical stiffness and elasticity of the hydrogels were tested and it was found that all hydrogels were relatively stiff, flexible, and mechanically elastic at the same time. The elasticity modulus of the hydrogels increased with increasing cross‐linking density. The OHA/Col‐CDH/MWCNT‐0.1 and OHA/Col‐CDH hydrogel showed the higher elastic modulus (>50 kPa), while the OHA/Col‐CDH/MWCNT‐0.2 hydrogel showed the suitable elastic modulus (38 kPa) (Figure 2h), which reached the range of native myocardial modulus (11.9–46.2 kPa),^[^ ^36^ ^]^ demonstrating that the hydrogel treatment system was suitable for use as a biological material to assist cardiac contraction. Considering the rheological properties, mechanical properties and electrical conductivity of the above four hydrogels, the OHA/Col‐CDH/MWCNT‐0.2 had the elastic modulus and electrical conductivity matching with natural myocardium, and also had self‐healing and injectable properties. Therefore, we chose OHA/Col‐CDH/MWCNT‐0.2 as the hydrogel material selected for subsequent in vivo and in vitro studies.

### 2.3 Anti‐Apoptosis, Antioxidant, and Mitochondria Protective Effect of the Hydrogel Treatment System

**Figure 3:**
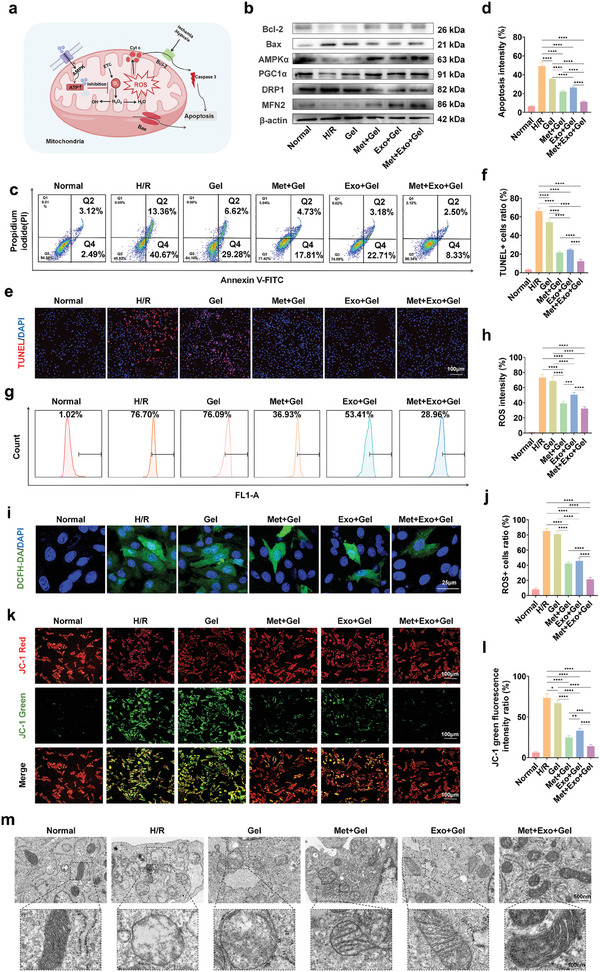
Effects of multifunctional hydrogel on anti‐apoptosis, antioxidant and mitochondria protection in vitro. a) Schematic diagram of the anti‐apoptotic, antioxidant, and mitochondria protection of the hydrogel system. b) Protein expression level of Bcl‐2, Bax, AMPKα, PGC1α, DRP1, and MFN2. c) Images of apoptosis flow cytometry analysis in each group. d) Quantitative analysis of apoptosis intensity in each group (flow cytometry). e) The TUNEL reaction images of each group were observed by fluorescence microscope. Scale bars: 100 µm. f) Quantitative analysis of TUNEL+ cells ratio in each group (fluorescence imaging). g) Images of DCFH‐DA flow cytometry analysis in each group. h) Quantitative analysis of ROS intensity in each group (flow cytometry). i) The DCFH‐DA probe images of each group were observed by confocal microscopy. Scale bars: 25 µm. j) Quantitative analysis of ROS cells ratio in each group (confocal imaging). k) Representative fluorescence images of the JC‐1‐stained mitochondrial membrane potentials of all groups. Scale bars: 100 µm. l) Quantitative analysis of JC‐1 green fluorescence intensity ratio values. m) TEM images of mitochondria following various treatments. Scale bars: 500 nm. The high‐magnification image is taken from the black box of the low‐magnification image. Scale bars: 100 nm. Statistical data by means ± SD (n = 5). One‐way ANOVA was used to compare multiple groups. *p < 0.05, **p < 0.01, ***p < 0.001, ****p < 0.0001.

Apoptosis and reactive oxygen species (ROS) production are important factors that exacerbate myocardial ischemia‐reperfusion injury. Reducing ROS production, inhibiting apoptosis, and protecting the structure and function of mitochondria is one of the important strategies to alleviate myocardial injury (**Figure** **3**a). We established an in vitro model of myocardial ischemia‐reperfusion using hypoxia/reoxygenation (H/R) methods and divided them into groups to evaluate this. Flow cytometry and fluorescence imaging were used to assess apoptosis and ROS production levels in the different treatment groups. As depicted in Figure 3c,d, treatment with Met+Gel and Exo+Gel significantly lowered the proportion of apoptotic cells, indicating a marked reduction in apoptotic and necrotic cells. Notably, the combination of metformin and exosomes exhibited a significant synergistic effect in inhibiting cardiomyocyte apoptosis, with the Met+Exo+Gel group demonstrating the most pronounced anti‐apoptotic effect observed in this study. We further verified the terminal deoxynucleotidyl transferase dUTP nick end labeling (TUNEL) experiment, and the fluorescence microscope images clearly showed that the apoptosis rate of H9c2 cells after metformin and exosome treatment was significantly reduced, as shown in Figure 3e,f. In addition, we further examined the expression of apoptosis‐related proteins. At the molecular level, the Met+Exo+Gel group demonstrated a significant upregulation of the anti‐apoptotic protein Bcl‐2 and a downregulation of the pro‐apoptotic protein Bax (Figure 3b). These results indicated that the combination of metformin and the exosome hydrogel treatment system effectively inhibited hypoxia‐induced apoptosis. Similarly, the Met+Gel and Exo+Gel groups also displayed notable anti‐apoptotic effects, but the combination exhibited superior efficacy. This enhanced effect was likely due to the synergistic actions of metformin and exosomes, including reducing ROS production and improving intracellular redox status. Based on this analysis, it can be concluded that Met+Exo+Gel effectively inhibited apoptosis and necrosis in H9c2 cells, thereby preventing further deterioration of MIRI and aiding in the restoration of heart functions.

The generation of a substantial amount of ROS during myocardial ischemia‐reperfusion is a critical mechanism contributing to myocardial injury.^[^ ^22^, ^37^ ^]^ Hence, controlling ROS production and eliminating excess ROS is a pivotal strategy to mitigate reperfusion injury and safeguard cardiomyocytes.^[^ ^38^ ^]^ In this study, an H/R model was employed to emulate the pathological process of myocardial ischemia‐reperfusion, thereby inducing significant ROS production. The results demonstrated that the hydrogel treatment system exhibited a potent antioxidant effect, as confirmed by the detection of ROS levels using dichlorodihydrofluorescein diacetate (DCFH‐DA) probes. Our findings revealed that DCFH‐DA fluorescence intensity significantly decreased with the administration of metformin and exosomes, with the most pronounced reduction observed when metformin and exosomes were combined within a hydrogel therapy system. Specifically, the DCFH‐DA fluorescence intensity in the Met+Exo+Gel group exhibited the greatest decrease among all groups, as evidenced by the leftward shift in the peak of its relative fluorescence intensity (Figure 3g,h). Confocal microscopy also showed a significant reduction in ROS production (green fluorescence) compared to the H/R group (Figure 3i,j). This shift suggested that the combination of metformin and exosomes had the most significant inhibitory effect on total ROS production. The combination therapy effectively reduced excessive oxidative stress in cells induced by hypoxia, thereby alleviating myocardial damage.

In order to further explore the mechanism of the hydrogel system to reduce MIRI, we conducted related experiments from the level of mitochondrial protection. A reduction in mitochondrial membrane potential (MMP) is a hallmark of early myocardial apoptosis. The MMPs of H9c2 cells subjected to various treatments were evaluated using a JC‐1 assay kit. Cells in the H/R groups demonstrated a marked increase in green fluorescence compared to the normal group, indicating significant mitochondrial dysfunction induced by H/R. However, this dysfunction was substantially ameliorated following treatment with metformin or metformin/MSC‐Exos‐loaded hydrogels, as reflected by a notable decrease in green fluorescence intensity (Figure 3k,l).

Mitochondrial damage caused by H/R is typically associated with pronounced structural alterations in mitochondria. TEM was employed to investigate these structural changes under different treatments. In the H/R group, cells exhibited morphological abnormalities, including swelling and irregular shapes, while mitochondria displayed severe swelling, disrupted cristae, and vacuolization. In contrast, cells treated with either metformin‐loaded hydrogels or metformin/MSC‐Exos‐loaded hydrogels showed significantly reduced mitochondrial swelling and vacuolization. These interventions effectively preserved the integrity of mitochondrial cristae and outer membranes (Figure 3m). The findings from both the JC‐1 assay and TEM analysis underscore the therapeutic potential of metformin, in mitigating mitochondrial damage and safeguarding mitochondrial structural integrity in myocardial cells.

To investigate the mechanisms underlying the cardioprotective effects of metformin and its ability to mitigate mitochondrial damage, we evaluated alterations in key molecular pathways using Western blot analysis. As a highly conserved energy sensor, AMPK plays a fundamental role in maintaining cellular energy balance and orchestrates a number of the therapeutic actions of metformin.^[^ ^7^ ^]^ Specifically, AMPK activation is critical for regulating mitochondrial biogenesis, with PGC1α acting as a principal downstream effector. Activation of AMPK promotes PGC1α activity, which subsequently facilitates the transcription of mitochondrial DNA and the expression of mitochondrial proteins.^[^ ^39^ ^]^ Results indicated a significant upregulation of AMPKα and PGC1α protein levels in the metformin‐treated groups (Figure 3b), suggesting that the observed protective effects against mitochondrial dysfunction during cardiac ischemia/reperfusion injury are mediated, at least partially, through the AMPK/PGC1α signaling pathway.

The role of metformin in modulating mitochondrial dynamics, a critical factor in MIRI, was also assessed. Dysregulation of mitochondrial dynamics, often characterized by excessive fission and fragmentation, exacerbates mitochondrial dysfunction during MIRI.^[^ ^40^ ^]^ The study demonstrated a notable increase in DRP1 expression in the H/R group relative to the normal group, indicating enhanced mitochondrial fission. Treatment with metformin significantly reduced DRP1 expression compared to the H/R group (Figure 3b), suggesting inhibition of aberrant fission. Additionally, a proper balance between mitochondrial fission and fusion is essential for preserving mitochondrial function. Promoting mitochondrial fusion, which prevents cellular death and heart failure, is facilitated by MFN2, a key fusion‐related protein.^[^ ^41^ ^]^ Metformin‐treated groups exhibited significantly increased MFN2 expression levels (Figure 3b), highlighting its role in restoring mitochondrial dynamics by reducing fission and enhancing fusion, thereby improving mitochondrial energy homeostasis. Interestingly, the expression of PGC1α, MFN2, and AKT was also upregulated to some extent in the Exo+Gel group, this may be related to the fact that the exosomes of mesenchymal stem cells contain a variety of bioactive molecules (such as miRNA, proteins and long‐chain non‐coding RNA, etc.) and may enhance intercellular communication, suggesting that the combination of exosomes and metformin is expected to further promote the structural and functional recovery of damaged mitochondria.

In summary, H/R injury disrupts the structural and functional integrity of cardiac mitochondria, leading to excessive production of ROS. This oxidative stress activates apoptotic pathways, ultimately resulting in cardiomyocyte damage. Metformin mitigates these detrimental effects by promoting mitochondrial biogenesis via the AMPK/PGC1α signaling pathway and modulating mitochondrial dynamics through the regulation of DRP1 and MFN2. These mechanisms contribute to the stabilization of mitochondrial structure, the restoration of mitochondrial function, and a subsequent reduction in ROS generation and apoptosis. Furthermore, exosomes appear to amplify the therapeutic effects of metformin on mitochondrial structure and function by releasing bioactive substances and enhancing intercellular communication, offering a synergistic approach to protecting cardiomyocytes from H/R‐induced injury.

### 2.4 The Effect of the Hydrogel Treatment System on Improving the Electrophysiological Properties of Cardiomyocytes

**Figure 4:**
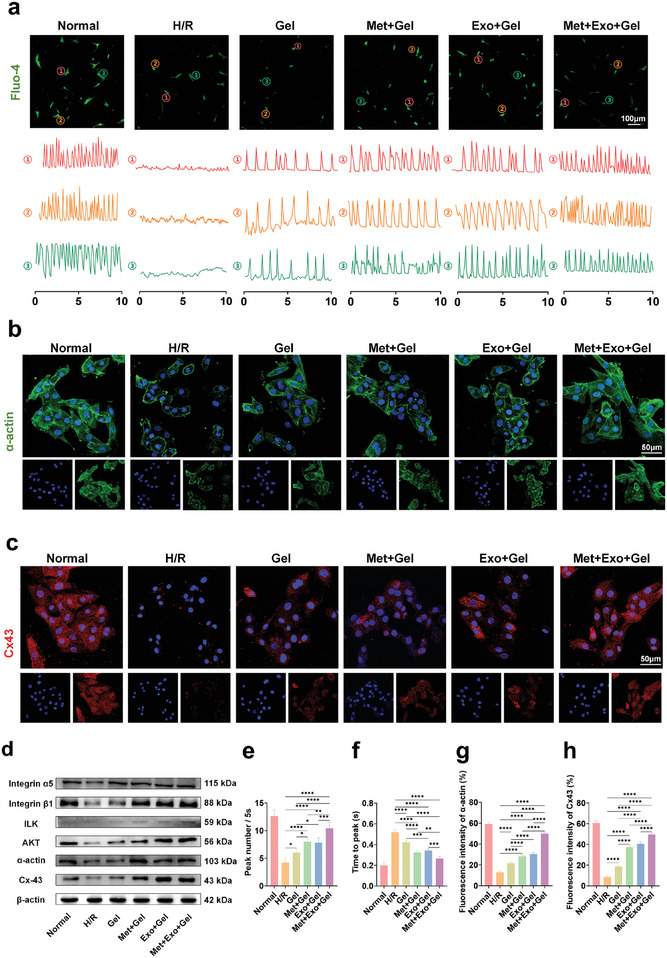
Effects of multifunctional hydrogel on improving electrophysiological properties of NRCMs in vitro. a) Examination of calcium transients and corresponding Ca^2+^ frequency signals in NRCMs from different experimental groups. Scale bars: 100 µm. b) Immunostaining of α‐actin in NRCMs from different experimental groups. Scale bars: 50 µm. c) Immunostaining of Cx43 in NRCMs from different experimental groups. Scale bars: 50 µm. d) Protein expression level of Integrinα5, Integrinβ1, ILK, AKT, α‐actin and Cx43. e) Quantitative analysis of calcium transient parameters focusing on peak frequency recorded every 5 seconds. f) Quantitative analysis of calcium transient parameters focusing on time to peak. g) Quantitative analysis of fluorescence intensity of α‐actin. h) Quantitative analysis of fluorescence intensity of Cx43. Statistical data by means ± SD (n = 5). One‐way ANOVA was used to compare multiple groups. *p < 0.05, **p < 0.01, ***p < 0.001, ****p < 0.0001.

Neonatal rat cardiomyocytes (NRCMs) exhibit rhythmic contractions and generate contractile forces driven by intracellular calcium signaling. In order to ascertain the impact of the conductive hydrogel on the contractile and electrophysiological performance of NRCMs, transient intracellular Ca^2^⁺concentrations were measured utilizing the fluorescent calcium indicator Fluo‐4. As shown in **Figure** **4**a, the H/R group displayed minimal spontaneous electrical activity and a lack of visible contractions. In contrast, treatment with the conductive hydrogel markedly improved transient Ca^2^^+^ propagation. Quantitative analysis of calcium transients indicated that the Met+Exo+Gel group exhibited a significantly shorter time to reach peak calcium levels and a higher frequency of calcium peaks (Figures 4e,f). These findings highlight the beneficial role of the conductive hydrogel in enhancing intracellular calcium dynamics. Furthermore, the combined action of metformin and MSC‐Exos within the hydrogel appears to facilitate the restoration of electrical signal conduction between cardiomyocytes, thereby improving myocardial function and demonstrating promising therapeutic potential.

α‐actin is a critical cardiac protein necessary for the maturation of cardiomyocytes and plays a key role in the reassembly of myofilaments in rat cardiomyocytes.^[^ ^42^ ^]^ Connexin 43 (Cx43), a primary connexin, is essential for the proper function of damaged myocardial cells, facilitating the formation of gap junctions that promote transmission of action potential and synchronous contraction between NRCMs.^[^ ^43^, ^44^ ^]^ To investigate the specific in vitro effects of the conductive hydrogel, we assessed the expression levels of α‐actin and Cx43 using both immunofluorescence and western blot analysis. As shown in Figure 4b,g, cardiomyocytes cultured on the conductive hydrogel displayed a greater density of α‐actin‐positive sarcomeres. Notably, the conductive hydrogel‐treated group exhibited strong Cx43 expression at the plasma membrane and between adjacent cells, which was absent in the H/R group (Figure 4c), a finding further supported by the fluorescence quantification data (Figure 4h). Western blot analysis confirmed a significant increase in α‐actin and Cx43 expression in the hydrogel‐treated groups compared to the H/R group (Figure 4d). This effect was particularly pronounced in the Met+Gel, Exo+Gel, and Met+Exo+Gel groups, aligning with the confocal microscopy observations. In conclusion, the incorporation of MWCNTs into the hydrogel significantly enhanced its electrical conductivity, which facilitated the coupling and synchronized contraction of cardiomyocytes. This improvement in electrical signal conduction between cells contributed to the recovery of myocardial function, highlighting the potential therapeutic benefits of this conductive hydrogel system.

Previous studies have established that integrins act as mechanosensors, linking the cytoskeleton to the extracellular matrix, and are essential for regulating electrical and mechanical junction proteins in cardiac cells, particularly under conditions of mechanical stress.^[^ ^45^ ^]^ Moreover, the β1 integrin signaling pathway has been shown to mediate cell‐matrix interactions, particularly those facilitated by conductive materials. In the present study, we explored the integrin‐mediated mechanotransduction pathway, focusing on key molecules such as integrin α5, integrin β1, Integrin‐linked kinase (ILK), and AKT. As illustrated in Figure 4d, the expression levels of both integrin α5 and β1 were significantly upregulated in the hydrogel‐treated group compared to the H/R group. Additionally, ILK is a crucial component of the sarcomeric contractile apparatus in the vertebrate heart.^[^ ^46^ ^]^ Through its interaction with integrins, ILK can activate downstream signaling pathways, including the AKT pathway, while AKT can regulate the expression of Cx43 in the heart.^[^ ^47^ ^]^ In our study, we observed a significant elevation in the levels of both ILK and AKT in the hydrogel‐treated group, especially in the Exo+Gel group and Met+Exo+Gel group, this may be attributed to the capacity of MSC exosomes to activate the PI3K/AKT pathway, which can collectively regulate AKT expression and, in turn, influence the expression of Cx43. These findings indicated that the integrin‐mediated electrophysiological conduction pathway was activated by the conductive hydrogel which can effectively promote the transmission of the action potential and synchronous contraction of the myocardium by the conduction characteristics obtained by incorporation of MWCNTs.

### 2.5 Promoting Migration and Tube Formation Effect of the Hydrogel Treatment System

**Figure 5:**
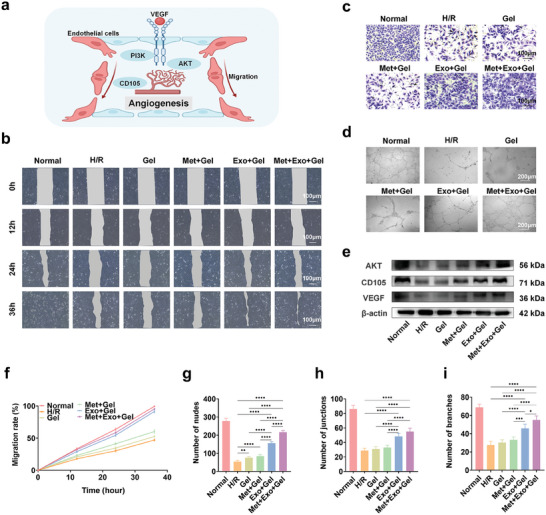
Effects of multifunctional hydrogel on promoting migration and angiogenesis in vitro. a) Schematic diagram of the promoting migration and angiogenesis of the hydrogel system. b) Representative images of HUVEC cultured with the component of the hydrogel system for 0, 12, 24, and 36 h. Scale bars: 100 µm. c) Transwell images of HUVEC cultured with the component of the hydrogels. Scale bars: 100 µm. d) Images of HUVEC forming tubes in vitro. Scale bars: 200 µm. e) Protein expression level of AKT, VEGF and CD105. f) Line chart of wound cell migration ratio at 12, 24, and 36 h. g–i) Quantitative results of the number of nodes (g), junctions (h), and branches (i) of different hydrogel groups. Statistical data by means ± SD (n = 5). One‐way ANOVA was used to compare multiple groups. *p < 0.05, **p < 0.01, ***p < 0.001, ****p < 0.0001.

Achieving effective repair of MIRI typically requires a robust angiogenic response, beginning in the marginal zone and extending to the core of the infarcted area.^[^ ^48^ ^]^ This response rapidly forms a dense capillary network essential for gas exchange, nutrient diffusion, and waste removal, thus meeting the high metabolic demands of the injure sites and preventing further cardiomyocyte death at the injure margin.^[^ ^49^ ^]^ To determine the functional role of this multifunctional hydrogel treatment system in cell migration and angiogenesis, we examined its effects on endothelial cells (**Figure** **5**a). That is, we built H/R models of HUVEC, and treated them accordingly in groups. The biological activity of hydrogels on HUVEC was assessed through various methods, including scratch assays, tube formation assays, and transwell assays, providing complementary insights into its angiogenic potential.

First, the effect of the hydrogel on endothelial cell migration was assessed. The scratch test is a simple, low‐cost in vitro assay of cell migration. As shown in Figure 5b, after 12 hours, significant cell proliferation and migration were observed in the hydrogel groups containing MSC‐Exos (Exo+Gel and Met+Exo+Gel groups). In contrast, the hydrogel groups without MSC‐Exos (Gel and Met+Gel groups) did not show a statistically significant difference compared to the control group. Moreover, by 36 hours, almost complete cell migration was seen in the hydrogel groups with MSC‐Exos, whereas the H/R group exhibited large non‐healing areas. These findings suggest that hydrogel‐loaded MSC‐Exos substantially enhance cell migration rates, which may be attributed to the role of MSC‐Exos in promoting endothelial cell proliferation and migration. Additionally, the quantitative cell migration rate data presented in Figure 5f align with these observations. On the other hand, transwell assay results demonstrated a greater promotion of cell migration in the Exo+Gel and Met+Exo+Gel group than other groups (Figure 5c and Figure S4, Supporting Information).

Endothelial cell vascularization is a crucial aspect of angiogenesis. To assess the angiogenic potential of the hydrogel treatment system, a tube formation assay was conducted through HUVEC. Following a 12‐hour incubation, significant angiogenesis was observed in the hydrogel‐loaded MSC‐Exos groups (Figure 5d). Quantitative analysis of the nodes, junctions, and branches from the tube formation images (Figure 5g–i) indicated that the hydrogel‐loaded MSC‐Exos group demonstrated a markedly higher capacity for tube formation in HUVEC compared to the H/R group and the hydrogel groups without MSC‐Exos. This finding aligns with previous studies showing that MSC‐Exos promotes angiogenesis in vitro. It is notable that the number of nodes and branches in the Met+Exo+Gel group was greater than that observed in the Exo+Gel group. This indicates that the combination of metformin and exosomes could facilitate angiogenesis in a more effective manner, potentially due to the role of metformin as an AMPK agonist. Metformin is capable of regulating cell energy balance and reducing oxidative stress, protecting endothelial cells from damage, and subsequently enhancing the function of exosomes in promoting angiogenesis.

CD105 functions as an accessory receptor for transforming growth factor beta (TGF‐β) and are up‐regulated in actively proliferating endothelial cells, rendering it an appropriate marker for neovascularization.^[^ ^50^, ^51^ ^]^ Similarly, **vascular endothelial growth factor (**VEGF) is a well‐known vascular permeability factor that facilitates endothelial cell proliferation and migration. Moreover, the AKT pathway plays a pivotal role in angiogenesis by regulating a series of processes associated with endothelial cell proliferation, migration, and survival, while also enhancing the transcription and secretion of VEGF.^[^ ^52^ ^]^ To assess the impact of the hydrogel on the expression levels of AKT, VEGF, and CD105, Western blot analysis was performed. As depicted in Figure 5e, the data indicated that the protein expression levels of AKT, CD105, and VEGF in HUVEC treated with hydrogel‐loaded MSC‐Exos (Exo+Gel and Met+Exo+Gel groups) were markedly higher than those in the control group. These results suggest that MSC‐Exos within the hydrogel system potentiate angiogenesis by enhancing the proliferation and migration of HUVEC and by up‐regulating the expression levels of CD105, AKT, and VEGF.

### 2.6 Exploring the Potential Mechanism of the Metformin/Exosome‐Loaded Hydrogel System through RNA Sequencing

**Figure 6:**
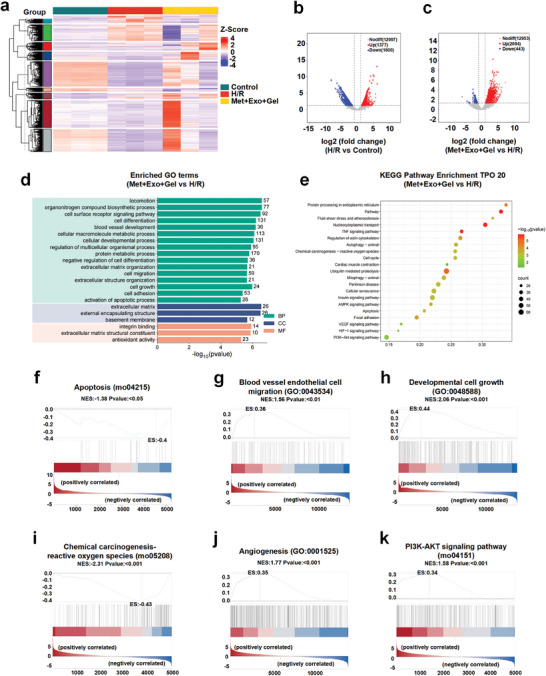
RNA sequencing was used to explore the potential mechanism of the hydrogel system. a) Heatmap of RNA‐seq expression levels of genes under different conditions. A total of three biological replicates were performed for each group. b) Volcano plots showing up‐ and down‐regulation of DEGs between Control and H/R. c) Volcano plots showing up‐ and down‐regulation of DEGs between Met+Exo+Gel and H/R. d) GO analysis of DEGs among Met+Exo+Gel versus H/R groups. e) KEGG pathway enrichment analysis of DEGs among Met+Exo+Gel versus H/R groups. f) GSEA of apoptosis signaling pathway. g) GSEA of blood vessel endothelial cell migration. h) GSEA of development cell growth. i) GSEA of chemical carcinogenesis‐reactive oxygen species. j) GSEA of angiogenesis. k) GSEA of PI3K‐AKT signaling pathway. The above GSEA was performed among Met+Exo+Gel versus H/R groups.

To further elucidate the transcriptional changes induced by treatment with a multifunctional hydrogel system containing metformin and exosomes, RNA sequencing (RNA‐seq) was performed on H9c2 cells subjected to H/R (**Figure** **6**a). As depicted in Figure 6b and Figure 6c, 2977 differentially expressed genes (DEGs) were identified in the H/R group compared to the control group, with 1377 upregulated and 1600 downregulated. 2527 DEGs were observed in the Met+Exo+Gel group compared to the H/R group, with 2084 upregulated and 443 downregulated. This indicates that the combination of metformin and exosomes significantly modulate the expression of a substantial number of genes.

Gene Ontology (GO) analysis revealed that the DEGs in the H/R group were predominantly associated with apoptotic processes and cell death, while downregulated genes were linked to the activation of cell cycle and chromosome segregation (Figure S5, Supporting Information). These findings confirm that H/R conditions promote cardiomyocyte apoptosis and death while inhibiting survival and proliferation. Conversely, in the Met+Exo+Gel group, gene enrichment analysis indicated significant associations with cell growth and antioxidant activity. Notably, upregulated genes were related to blood vessel development, cell migration, and cell growth (Figure 6d).

Moreover, Kyoto Encyclopedia of Genes and Genomes (KEGG) functional enrichment analysis revealed that the upregulated genes in the H/R group were implicated in several canonical signaling pathways, including mTOR, TGF‐β, apoptosis, and reactive oxygen species pathways (Figure S6, Supporting Information). The activation of these pathways leads to impaired cardiomyocyte function and exacerbates cardiac pathology, thereby confirming the role of H/R in simulating the pathophysiological processes associated with myocardial ischemia and cardiac dysfunction. In contrast, the upregulated genes in the Met+Exo+Gel group were found to be enriched in pathways related to AMPK, autophagy, cell cycle, and VEGF signaling, while the downregulated genes were primarily associated with apoptosis and reactive oxygen species pathways (Figure 6e). This differential gene expression pattern may be attributed to the synergistic effect of metformin and exosomes, which together activate the AMPK and VEGF pathways, effectively reduce the levels of ROS and apoptosis, and promote angiogenesis based on the maintenance of mitochondrial energy metabolism, which would be beneficial for myocardial injury repair, and is consistent with the experimental results in the previous parts of this study.

Gene set enrichment analysis (GSEA) was conducted to explore the mechanistic basis for the reduction of MIRI by the multifunctional hydrogel system. The enriched genes primarily focused on pathways involved in apoptosis, oxidative stress, and angiogenesis, including the apoptosis signaling pathway (Figure 6f), endothelial cell migration (Figure 6g), developmental cell growth (Figure 6h), chemical carcinogenesis‐reactive oxygen species (Figure 6i), angiogenesis (Figure 6j), PI3K‐AKT (Figure 6k), AMPK (Figure S7, Supporting Information), and VEGF (Figure S8, Supporting Information) signaling pathway, which was consistent with the experimental results in the previous parts of this study. Collectively, the RNA‐seq data provided compelling evidence that the hydrogel system containing metformin and exosomes enhances cardiomyocyte proliferation and repair, as well as improving cardiac function through multiple mechanisms, such as anti‐apoptotic, antioxidant, pro‐angiogenic, and AMPK pathway activation. These findings offered a bioinformatic basis for the potential therapeutic application of this hydrogel system in treating MIRI.

### 2.7 Evaluation of Therapeutic Effects of Multifunctional Hydrogels Treatment System In Vivo

**Figure 7:**
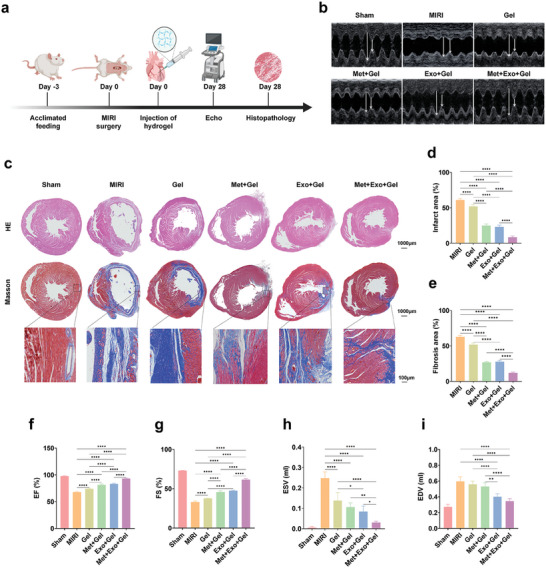
Multifunctional hydrogels attenuate myocardial infarction and fibrosis areas and improve cardiac function. a) Schematic showing the timeline for MIRI induction, hydrogel injection, cardiac functional evaluation, and pathological examination over the course of 28 days. b) Representative echocardiographic images of each group. c) HE and Masson staining of the infarcted areas. Scale bars: 1000 µm. The high‐magnification image is taken from the black box of the low‐magnification image. Scale bars: 100 µm. d,e) Quantitative analysis of infarct size (d) and fibrosis area (e) in each group after treatment. f–i) Cardiac function indicators were determined by echocardiography after treatment, including EF (f), FS (g), ESV (h), and EDV (i). Statistical data by means ± SD (n = 5). One‐way ANOVA was used to compare multiple groups. *p < 0.05, **p < 0.01, ***p < 0.001, ****p < 0.0001.

To assess the therapeutic impact of hydrogels on MIRI in vivo, a MIRI model was created by removing the ligature after 1 hour of ligation of the left anterior descending artery (LAD) in rats (Figure S9, Supporting Information), then the successful MIRI model construction would be confirmed by typical electrocardiograph (ECG) (Figure S10, Supporting Information). The rats were randomly assigned to six groups: Sham, MIRI, Gel, Met+Gel, Exo+Gel, and Met+Exo+Gel. Four weeks after hydrogel injection, the cardiac function of rats was evaluated by ultrasonic M‐mode in small animals, and then the hematoxylin‐eosin and Masson staining were used to evaluate treatment efficacy (**Figure** **7**a).

Echocardiography showed that there were some differences in cardiac function among the experimental groups (Figure 7b). The functional evaluation of this research center is mainly based on the following parameters: ejection fraction (EF), fractional shortening (FS), left ventricular end‐diastolic dimension (LVIDd), left ventricular end‐systolic dimension (LVIDs), end‐diastolic volume (EDV), and end‐systolic volume (ESV) (Figure 7f–i and Figures S11 and S12, Supporting Information), echocardiography revealed a significant decrease in EF and FS, alongside a notable increase in LVIDs, LVIDd, ESV, and EDV in the MIRI group compared to the sham group, indicating severe cardiac dysfunction and left ventricular remodeling in the rats in the MIRI group. In contrast, rats treated with the hydrogel exhibited higher EF and FS and lower LVIDd, LVIDs, EDV, and ESV measurements than the MIRI group. Specifically, all groups containing hydrogel showed promising therapeutic effects on MIRI, likely attributable to the mechanical support provided by the hydrogel and the electrical conductivity conferred by MWCNT. Furthermore, both EF and FS were significantly improved in the Met+Gel and Exo+Gel groups compared to the Gel group, underscoring the therapeutic contributions of metformin and MSC‐Exos in myocardial infarction tissue repair. Notably, the Met+Exo+Gel group demonstrated the most pronounced therapeutic benefits, suggesting a synergistic effect of metformin and MSC‐Exos in enhancing cardiac function and mitigating infarct expansion. These results emphasized the efficacy of the multifunctional hydrogel therapy system loaded with metformin and MSC‐Exos in improving cardiac performance by reducing infarct size and increasing left ventricular wall thickness in the infarcted area.

Next, histological analyses were performed to investigate the long‐term impact of multifunctional hydrogel treatment on cardiac remodeling after MIRI. Consistent with the improvement in cardiac function, cardiac morphology was also improved by the hydrogel treatment system. The MIRI group exhibited severe myocardial infarction presentation indicated by myocardial collapse, wall thinning, infarct fibrosis, and extensive blue collagen deposition (Figure 7c). Quantitative analysis (Figure 7d,e) revealed that all hydrogel‐treated groups showed increased ventricular wall thickness and reduced collagen deposition compared to the MIRI group. This improvement was likely due to the mechanical support provided by the hydrogel, which helped to withstand myocardial stress. Additionally, the presence of MWCNT in the hydrogel aided in restoring physiological electrical activity in the infarct area, enhancing the microenvironment and preventing further infarct expansion. Notably, the group treated with hydrogel containing metformin and exosomes exhibited a significant increase in left ventricular infarct wall thickness. The Met+Exo+Gel group showed the greatest wall thickness and smallest infarct area at 28 days, likely due to the synergistic effects of metformin and exosomes in combating ROS and apoptosis.

### 2.8 Effects of Multifunctional Hydrogel on Anti‐Apoptosis, Promoting Cell Proliferation and the Expression of Cardiac‐Specific Markers In Vivo

**Figure 8:**
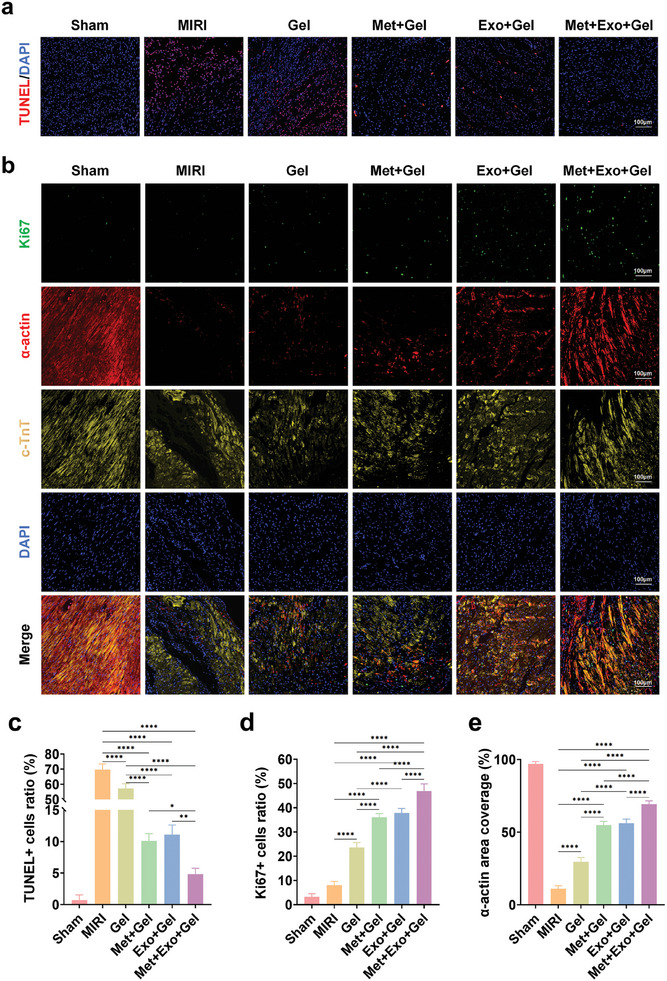
Effects of multifunctional hydrogel on anti‐apoptosis, promoting cell proliferation and the expression of cardiac‐specific markers in vivo. a) Representative TUNEL staining images of the injury area through different treatments after 3 days. Scale bars: 100 µm. b) Immunofluorescence images of Ki67, α‐actin, and c‐TnT trichrome staining in the injury area through different treatments after 4 weeks. Scale bars: 100 µm. c–e) Quantitative analysis of TUNEL (c), Ki67 (d), and α‐actin (e). Statistical data by means ± SD (n = 5). One‐way ANOVA was used to compare multiple groups. *p < 0.05, **p < 0.01, ***p < 0.001, ****p < 0.0001.

In the pathophysiology of MIRI, a large amount of reactive oxygen species will be produced in the myocardial microenvironment which the body cannot entirely neutralize. We initially examined if the hydrogel could effectively lower the elevated ROS levels post‐MIRI in vivo. In this research, the ROS fluorescence signal and relative fluorescence intensity were notably higher in the MIRI group compared to the sham group, indicating an increase in ROS levels post‐MIRI. Conversely, the Met+Gel and Met+Exo+Gel groups displayed weaker fluorescence signals and lower relative fluorescence intensities (Figures S13 and S14, Supporting Information), suggesting that metformin effectively reduces ROS levels at the damaged site, primarily due to its antioxidant properties. If ROS levels are not adequately mitigated, the excessive ROS can lead to cardiomyocyte apoptosis. Consequently, cardiomyocyte apoptosis was evaluated using the TUNEL staining method. As anticipated, all hydrogel treatment groups demonstrated a reduction in cardiomyocyte apoptosis at 3 days. Notably, the Met+Gel group, the Exo+Gel group, and the Met+Exo+Gel group exhibited a substantial reduction in apoptosis compared to the MIRI group (**Figure** **8**a,c), especially in the Met+Exo+Gel group.

The synchronous contractility of cardiomyocytes hinges on the expression of α‐actin, which plays a crucial role in myocardial maturation and the regulation of muscle contraction. This protein is essential for maintaining the structural integrity and functionality of cardiomyocytes, which is often used as a reference index to assess cardiac function. To investigate the relationship between cardiomyocyte proliferation and recovery of cardiac function, we performed immunofluorescence co‐localization experiments using cTnT (a cardiomyocyte‐specific marker), Ki67 (a proliferation marker), and α‐actin (a structural marker of myocardial segments). Analysis showed that some cTnT‐positive cells also expressed Ki67, indicating that these cardiomyocytes were in a proliferative state. Notably, α‐actin expression was observed in these Ki67‐positive cardiomyocytes, with the area of α‐actin expression positively correlating with the number of Ki67+ cells. For example, the expression levels of Ki67 and α‐actin were significantly higher in the Met+Gel, Exo+Gel, and Met+Exo+Gel groups than in the MIRI group (Figure 8b,d,e), suggesting that the formation of myofibrillar structures occurred in association with cardiomyocyte proliferation. These results suggest that metformin and exosomes promote the expression of markers associated with cardiomyocyte proliferation, potentially contributing to structural and functional remodeling of the myocardium. However, it is important to note that improvements in myocardial function are likely to result from the synergistic effects of multiple mechanisms, although proliferation is a promising indicator of potential regeneration, further studies are needed to definitively establish the causal role of cardiomyocyte proliferation in the recovery of cardiac function.

### 2.9 Evaluation of the Effects of Multifunctional Hydrogels on Improving Myocardial Conduction and Reducing the Susceptibility to Ventricular Arrhythmias In Vivo

**Figure 9:**
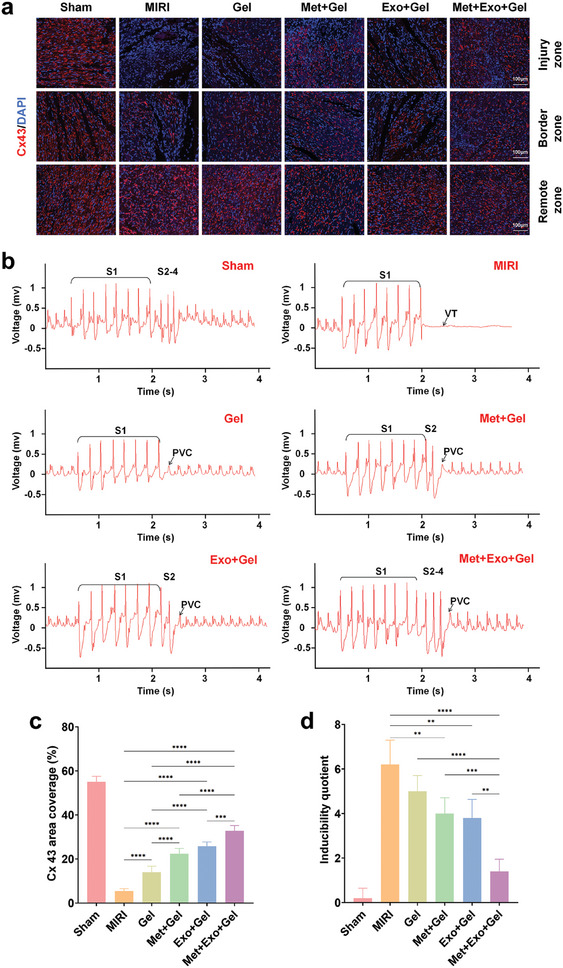
Effects of multifunctional hydrogel on improving myocardial conduction and reducing the susceptibility to ventricular arrhythmias in vivo. a) Representative Cx43 staining images of the injury, border, and remote area through different treatments after 28 days. Scale bars: 100 µm. b) ECGs of arrhythmias induced by PES at 4 weeks post‐injection. c) Quantitative analysis of Cx43 in injury area. d) Arrhythmia susceptibility determined by the inducibility quotient. S1: 8 burst stimulations; S2: single extra‐stimulus; S3: double extra‐stimuli; S4: triple extra‐stimulus. VT: ventricular tachycardia; PVC: premature ventricular contraction. Statistical data by means ± SD (n = 5). One‐way ANOVA was used to compare multiple groups. *p < 0.05, **p < 0.01, ***p < 0.001, ****p < 0.0001.

Cx43, is responsible for electrical coupling within the myocardium, enabling synchronized cardiac muscle contraction necessary for efficient heart function. To assess the potential of conductive hydrogels to enhance electrical signal transduction in vivo, myocardial tissue samples were subjected to immunofluorescence staining for Cx43. As shown in **Figure** **9**a,c, the expression of Cx43 was significantly elevated in the injury regions of hydrogel‐treated groups compared to the MIRI group, with the most substantial upregulation observed in the Met+Exo+Gel group, this finding underscored the superior therapeutic efficacy of the combination therapy, which leverages the synergistic effects of metformin and MSC‐Exos. This increased expression of Cx43 suggests enhanced electrical conduction and improved synchronization of cardiomyocyte contractions, both of which are crucial for the recovery of normal cardiac function.

The ability of conductive hydrogels to reduce the susceptibility of damaged hearts to sustained ventricular arrhythmias was also evaluated. Four weeks after treatment, all rats underwent programmed electrical stimulation (PES), a standard clinical protocol for arrhythmia induction. In the MIRI group, persistent ventricular tachycardia (VT) occurred immediately after the S1 cycle stimulation. In contrast, the majority of hydrogel‐treated groups exhibited premature ventricular contraction (PVC) only after stimulation, particularly the Exo+Met+Gel group, which demonstrated non‐persistent PVC on occasion after S4. These findings indicated that rats treated with hydrogels exhibited a significantly lower induction quotient compared to the MIRI group, reducing arrhythmia susceptibility (Figure 9b,d). This improvement reflects enhanced electrical activity in the myocardial tissue. The observed improvement in electrical conduction can be attributed to the high conductivity of MWCNTs, which facilitated effective signal transmission across the damaged myocardium. By partially reconstructing the conductive network between the damaged area and adjacent healthy tissue, the hydrogel promoted the rapid propagation of electrical signals from the functional myocardium to electrically isolated cardiomyocytes. This process effectively mitigated the risk of ventricular arrhythmias, marking an important step toward restoring the electrical integrity and functionality of damaged cardiac tissue.

### 2.10 Evaluation of the Effects of Multifunctional Hydrogels on Angiogenesis In Vivo

**Figure 10:**
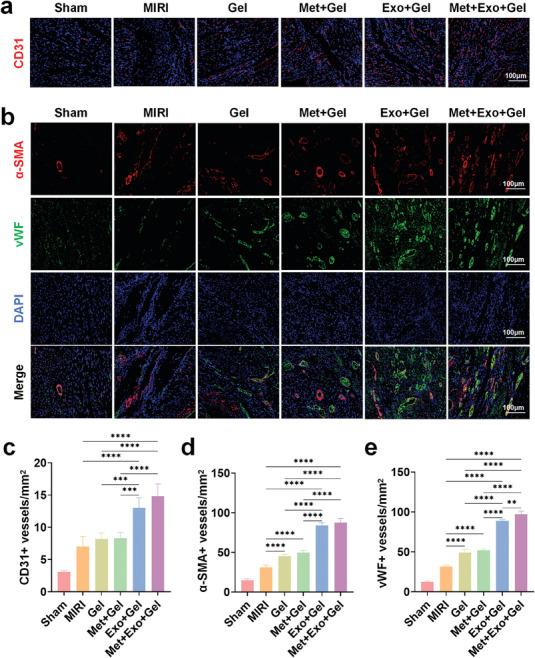
Evaluation of the effects of multifunctional hydrogels on angiogenesis in vivo. a) Immunofluorescence images of CD31 staining in the injury area through different treatments after 4 weeks. Scale bars: 100 µm. b) Immunofluorescence images of α‐SMA and vWF double staining in the injury area through different treatments after 4 weeks. Scale bars: 100 µm. c‐e) Quantitative analysis of CD31 positive vessels (c), α‐SMA‐positive vessels (d), and vWF‐positive vessels (e) in injury regions. Statistical data are expressed as mean ± SD (n = 5). One‐way ANOVA was used to compare multiple groups. *p < 0.05, **p < 0.01, ***p < 0.001, ****p < 0.0001.

Following myocardial injury, the revascularization of the damaged myocardium is paramount for the restoration of cardiac function. Enhancing angiogenesis serves as an effective strategy to reestablish the supply of oxygen and nutrients to the affected region. To assess angiogenesis, cardiac tissue sections were stained with α‐SMA (marking mature arterioles).^[^ ^53^ ^]^ vWF (marking microvessels).^[^ ^54^ ^]^ and CD31 (marking endothelial vessels).^[^ ^55^ ^]^ As depicted in **Figure** **10**a,b, 4 weeks post‐treatment, minimal angiogenesis was observed in the MIRI group, likely reflecting active cardiac compensation mechanisms. In stark contrast, the hydrogel treatment groups exhibited enhanced formation of new blood vessels, with the hydrogel containing MSC‐Exos demonstrating the highest degree of angiogenesis. This pronounced effect is attributed to the pro‐angiogenic properties of MSC‐Exos, aligning with the results from in vitro experiments. Quantitative analyses of CD31 (Figure 10c), α‐SMA (Figure 10d), and vWF (Figure 10e) labeled blood vessel density further substantiated the fluorescence staining results. These findings underscored the efficacy of the multifunctional hydrogel in promoting cardiac angiogenesis. Therefore, this hydrogel treatment system not only facilitated cell proliferation but also aided in the recruitment of endothelial cells to the damaged area, presenting a promising therapeutic approach for MIRI.

### 2.11 Biocompatibility and Cell Proliferation of Multifunctional Hydrogels

Calcein‐AM (live)/PI (dead) staining assays (Figures S15 and S16, Supporting Information) revealed that most cells treated with the hydrogels remained viable (green fluorescence), with very few dead cells (red fluorescence), and exhibited a healthy cell morphology. With the prolongation of time, the number of viable cells in the field of view gradually increased, indicating that the hydrogel had good cytocompatibility and promoted cell proliferation. Following this, the cytocompatibility of the hydrogels was tested using HUVEC and H9c2 cells. Cell viability assays at 24, 48, and 72 h (Figures S17 and S18, Supporting Information) showed that the hydrogels, including their drug‐loaded versions, did not negatively impact the viability of either cell type compared to the control group, indicating good cytocompatibility. Additionally, hemolysis testing is critical for evaluating the safety of medical device materials and products that directly or indirectly contact blood. The in vitro hemolysis results shown in FigureS19 (Supporting Information), indicated that all hydrogels had a hemolysis ratio of about 0.5%, well below the clinical threshold of 5%.^[^ ^56^ ^]^ This low hemolysis ratio demonstrated the excellent hemocompatibility of hydrogels. Overall, these findings confirmed that the hydrogels possessed excellent biological safety, as evidenced by their low hemolysis ratios and high cytocompatibility. Furthermore, on day 28, internal organs were stained with hematoxylin and eosin (HE), and no significant inflammatory effects were observed (Figure S20, Supporting Information), indicating that the hydrogel treatment system had acceptable biocompatibility.

## 3 Conclusion

In this study, a novel injectable conductive hydrogel loaded with metformin/MCS‐Exos was developed based on the pathological microenvironmental characteristics of MIRI, achieving on‐demand release of metformin and exosomes in the weakly acidic ischaemic microenvironment of the myocardium. The hydrogel exhibited a stable three‐dimensional cross‐linked structure, excellent biocompatibility, and injectable, and also closely matched the electrical conductivity and elastic modulus of natural cardiac tissue. The combined effect of metformin and exosomes released on demand has demonstrated remarkable efficacy in both in vivo and in vitro experimental studies. In vitro experiments demonstrated that the multifunctional hydrogel, when loaded with metformin and MSC‐Exos, synergistically protected mitochondrial structure and function, reduced cellular ROS levels, mitigated apoptosis, promoted cell migration and angiogenesis, improved the electrophysiological characteristics and exhibited excellent biocompatibility. Meanwhile, the transcriptome RNA sequencing showed that the hydrogel system was able to effectively activate the PI3K/AKT, VEGF, and AMPK signaling pathways, providing bioinformatics support for confirming its anti‐apoptosis and pro‐angiogenesis ability. Subsequent in vivo studies confirmed the efficacy of this hydrogel delivery system in reducing infarct size, cardiac fibrosis, and arrhythmia incidence, improving ventricular ejection fraction, and upregulating the expression of key cardiac markers, including α‐actin, Cx43, Ki67, and α‐SMA, and downregulating the expression of TUNEL, ultimately leading to improved cardiac function after MIRI. In conclusion, we have proposed the development of an injectable pH‐responsive conductive hydrogel with the capacity to facilitate the intelligent release of drugs (metformin) and bioactive substances (exosomes) in order to enhance cardiac repair. This represents a promising and novel therapeutic approach for the treatment of MIRI.

## 4 Experimental Section

### Preparation of OHA

The synthesis of OHA was conducted in accordance with a previously established methodology.^[^ ^57^ ^]^ Initially, 4.0 g of HA was solubilized in 100 mL of deionized water. Thereafter, 1.08 g of sodium periodate was added to the HA solution and stirred for 24 h at room temperature. Subsequently, 0.5 mL of ethylene glycol was added to the aforementioned solution, which was then stirred for a further 2 h. Finally, the mixture was subjected to dialysis and freeze‐drying, resulting in the production of OHA.

### Preparation of Col‐CDH

The synthesis of Col‐CDH was conducted in accordance with a previously established methodology.^[^ ^27^ ^]^ Initially, 4.0 g of Col was solubilized in 500 mL of deionized water. Upon complete dissolution of the collagen, 6.0 g of Carbohydrazide (CDH) was added to the aforementioned solution and continuously stirred. Subsequently, 4.0 g of 1‐(3‐Dimethylaminopropyl)‐3‐ethylcarbodiimide hydrochloride (EDC) and 1.2 g of N‐Hydroxy succinimide (NHS) were introduced to the gelatin solution, and the pH was adjusted to 4.8 mixing overnight. Subsequently, the mixture was subjected to dialysis and freeze‐drying, resulting in the formation of Col‐CDH.

### Preparation of the Hydrogel System

Exosomes extracted from mesenchymal stem cells (MSCs) were diluted to a concentration of 100 µg mL^−1^ using phosphate‐buffered saline (PBS). OHA was then dissolved in the exosome‐containing PBS to achieve a 10 wt% (w/v) concentration, followed by dispersing MWCNT in this solution. Concurrently, a stock solution of Col‐CDH at a 10 wt% (w/v) concentration was prepared in PBS (pH 7.4), and metformin hydrochloride (4 mg mL^−1^) was dissolved in this mixture. The hydrogel matrix was formed by combining equal volumes of the OHA/exosomes solution with varying proportions of MWCNT and the Col‐CDH/metformin solution at room temperature. Detailed compositions of all hydrogels are listed in Table S1 (Supporting Information). The hydrogels were designated OHA/Col‐CDH, OHA/Col‐CDH/MWCNT‐0.1, OHA/Col‐CDH/MWCNT‐0.2, and OHA/Col‐CDH/MWCNT‐0.5 based on their MWCNT content. The composition of the hydrogels was verified using Fourier‐transformed infrared spectroscopy (Spectrum Two, Perkin Elmer, USA) and ^1^H nuclear magnetic resonance (^1^H NMR, Avance II 300 MHzNMR, Bruker, Germany). Gelation time was determined using the inverted bottle method at 37 °C, and cross‐sectional morphology was analyzed via scanning electron microscopy (SEM) (JSM‐7500F, JEOL, Japan).

### Hypoxia/Reoxygenation (H/R) Cell Model and Grouping

H9c2 and HUVEC Cells (Chinese Academy of Science Cell Bank, China) were cultured in Dulbecco's Modified Eagle Medium (DMEM, Gibco, USA) with 10% fetal bovine serum and 1% penicillin‐streptomycin solution, maintained at 37 °C in a humidified atmosphere of 5% CO_2_. The culture media was renewed every 2 days and switched to minimal essential serum‐free medium 12 hours before treatment. For the HR model, H9c2/HUVEC cells were first placed in a hypoxic buffer (1% O_2_, 5% CO_2_, 94% N_2_) for 2 hours to remove residual oxygen. The cells were then transferred to a portable cell culture device, continuously supplied with the hypoxic gas mixture, and kept in a 37 °C incubator with saturated humidity for 6 hours of hypoxic treatment. Subsequently, the buffer was replaced with serum‐free medium, and the cells were reoxygenated for 2 hours under normal oxygen conditions. The cells were randomly divided into the following treatment groups: 1) Normal group, 2) H/R group, 3) Gel group, 4) Met+Gel group, 5) Exo+Gel group, and 6) Met+Exo+Gel group. All groups were treated with H/R except Normal group.

### Western Blot Analysis

Cellular proteins were separated using sodium dodecyl sulfate–polyacrylamide gel electrophoresis and then transferred to polyvinylidene fluoride membranes. The membranes were blocked using 5% nonfat dry milk for 2 h and then incubated with primary α‐actin (11313‐2‐AP, Proteintech), Connexin 43 (26980‐1‐AP, Proteintech), CD105 (10862‐1‐AP, Proteintech), VEGF(19003‐1‐AP, Proteintech), BAX(50599‐2‐Ig, Proteintech), Bcl‐2(68103‐1‐Ig, Proteintech), Integrin β1(12594‐1‐AP, Proteintech), Integrin α5(10569‐1‐AP, Proteintech), ILK(12955‐1‐AP, Proteintech), AKT(80816‐1‐RR, Proteintech), AMPKα(10929‐2‐AP, Proteintech), PGC1α(66369‐1‐Ig, Proteintech), DRP1(12957‐1‐AP, Proteintech) and MFN2(12186‐1‐AP, Proteintech) antibodies at 4 °C overnight. β‐actin was used as a control. After washing thrice, the membranes were incubated with horseradish peroxidase‐conjugated secondary anti‐rabbit IgG antibodies (1:3000, Boster, Wuhan, China) for 1 h at 37 °C. ECL detection system was used to measure the intensity of protein expression.

### MIRI Rat Model and Grouping

All animal procedures were approved by the Animal Research Committee of Jinan University (IACUC‐20240108‐07). Male Sprague–Dawley rats, weighing 220 ± 20 g, were obtained from Guangdong Weitonglihua Laboratory Animal Technology. To induce the MIRI model, the rats were anesthetized with 2% isoflurane, and their tracheas were intubated and connected to a ventilator. A left thoracotomy was performed to expose the heart, and the left anterior descending coronary artery was ligated with a 6‐0 suture. One hour after inducing ischemia, the suture was removed to allow reperfusion. The successful induction of MIRI was confirmed by observing a whitish left ventricular myocardium and typical ST‐segment elevation on an electrocardiograph. The hydrogels were injected into the left anterior wall immediately after MIRI was confirmed. According to the group assignments, 100 µL of the respective hydrogels or PBS was injected intramyocardially at the injury border using a 30‐gauge needle. The sham group underwent thoracotomy without coronary artery ligation. They were provided with a standard pellet diet and water ad libitum. The rats were randomly divided into the following treatment groups: 1) Sham group (n = 6, 100 µL PBS), 2) MIRI group (n = 6, 100 µLPBS), 3) Gel group (n = 6, 100 µL hydrogel), 4) Met+Gel group (n = 6, 100 µL hydrogel containing 100 µg metformin), 5) Exo+Gel group (n = 6, 100 µL hydrogel containing 50 µg MSC‐exo), and 6) Met+Exo+Gel group (n = 6, 100 µL gel containing 100 µg metformin and 50 µg MSC‐exo).

### Statistical Analysis

All data were expressed as mean ± standard deviation. Sample sizes are detailed in the figure captions. Statistical analyses were conducted using GraphPad Prism software. One‐Way Analysis of Variance (ANOVA) and Student's *t*‐test were utilized to determine statistical significance, with a *p*‐value of less than 0.05 considered significant.

## Conflict of Interest

The authors declare no conflict of interest.

## Supporting information



Supporting Information

## Data Availability

The data that support the findings of this study are available from the corresponding author upon reasonable request.
