# Supplementary material for: Injectable pH Responsive Conductive Hydrogel for Intelligent Delivery of Metformin and Exosomes to Enhance Cardiac Repair after Myocardial Ischemia‐Reperfusion Injury
Source: Adv Sci (Weinh). 2025 Feb 18;12(24):2410590. doi: 10.1002/advs.202410590 (PMC12199616; doi:10.1002/advs.202410590)
Supplement: Supplementary file 1 — Supporting Information [file ADVS-12-2410590-s001.docx]

Supporting Information

Injectable pH Responsive Conductive Hydrogel for Intelligent Delivery of Metformin and Exosomes to Enhance Cardiac Repair After Myocardial Ischemia-Reperfusion Injury

Nianlan Cheng^#^, Qiao Luo^#^, Yongqing Yang, Ni Shao, Tianqi Nie, Xiujiao Deng, Jifeng Chen, Siqi Zhang, Yanyu Huang, Kuan Hu*, Liangping Luo*, Zeyu Xiao*.

1. Materials

Hyaluronic acid (HA) and collagen (Col) was purchased from Maikelin Biotechnology Co., Ltd. Metformin hydrochloride  was obtained from Sigma-Aldrich. Carbohydrazide and carboxylated multi-walled carbon nanotubes (MWCNT) with 20–30-nm diameter, 10–30-μm length, were obtained from Shanghai Merrier Biochemical Technology Co., LTD. All reagents were of analytical grade and used directly without further purification. Calcein-AM/PI Double Staining Kit was obtained from KEYGEN BIOTECH (Jiangsu, China). Annexin V-FITC/PI Double Staining Kit, DCFH-DA Staining Kit and Mitochondrial membrane potential assay kit with JC-1 were obtained from Beyotime (Shanghai, China). CCK8 Kit was obtained from MeilunBio (Dalian, China). TUNEL Apoptosis Assay Kit Lablead (Beijing, China).

2. Methods

**2.1****.** **Characterization of hydrogels**

2.1.1. Rheological properties of hydrogels

The rheological properties of different hydrogels were evaluated at 37 °C. Specifically, 500μL samples of hydrogels were loaded onto the rheometer. The upper plate was set to a 1 mm gap to measure the storage modulus (G′) and loss modulus (G″) over a 15-minute period at a frequency of 1 Hz. Shear-thinning properties were examined across a shear rate range from 1 s⁻¹ to 100 s⁻¹. Strain sweeps, varying from 0.1% to 1000%, were conducted to determine the critical strain region where G′ and G″ intersect. The self-healing ability of the hydrogels was tested by applying three cycles of step strain from 1% to 500%.

2.1.2. Mechanical properties of hydrogels

The mechanical properties of the hydrogels were evaluated using a Universal Materials Tester. Cylindrical samples, each about 10 mm in height and 12 mm in diameter, underwent compression tests at room temperature with a constant speed of 5 mm/min. The elastic moduli was calculated by analyzing the initial slope of the compression curves within the elastic region.

2.1.3. Electrochemical properties of hydrogel

To assess the electrochemical properties of the hydrogels, two distinct techniques were employed. The conductivity of hydrogel flakes (1 cm in diameter) was measured using the standard four-point probe method (FM100GH, YAOS China). Cyclic voltammetry analyses were conducted at room temperature with an electrochemical workstation (CHI660E, CH, China). For these tests, a hydrogel-coated glassy carbon electrode was used as the working electrode, a Hg/Hg₂Cl₂ electrode as the reference, and a platinum electrode as the counter electrode, with 0.1 M PBS as the electrolyte. The voltage range was set between -0.6 and 0.8 V, with a sweep rate of 100 mV/s and a frequency range from 1 MHz to 0.01 Hz.

**2.2. Isolation of bone marrow mesenchymal stem cells**

Rat bone marrow mesenchymal stem cells (MSCs) were isolated from the femurs of 2-week-old Sprague-Dawley rats according to the previous literature^[1]^. Dulbecco's Modified Eagle Medium (DMEM) supplemented with 10% fetal bovine serum (FBS) (Gibco, USA) containing bone marrow cells was cultured at 37 °C, 5% CO2 cell culture incubator. The medium was replaced for the first time after 24–48 h and was replaced every 3 days afterward. Passage 3–5 (P3–P5) cells were used for subsequent experiments.

**2.3. Extraction and characterization of** **mesenchymal stem cell exosomes (MSC-Exos)**

MSCs were cultured in DMEM containing 10% exosome-free FBS for 48 h to collect conditioned medium. Centrifuge the harvested media at 2000 × g for 30 minutes to remove cells and debris. Exosomes Isolation reagent(invitrogen, USA) was used to isolate the exosomes of BMSC. Ensure thorough mixing by vortexing or pipetting until the solution is homogeneous. Incubate the mixture overnight at a temperature of 2°C to 8°C. Following the incubation period, centrifuge the samples at 10,000 × g for 1 hour at 2°C to 8°C. Carefully aspirate and discard the supernatant, retaining the pellet at the bottom of the tube, which contains the exosomes. Resuspend the pellet in an appropriate volume of 1xPBS or a similar buffer. The BCA method was employed to quantify the protein content of exosomes. Western blot analysis was conducted to identify exosomes biomarkers such as CD63 and TSG101. Transmission electron microscope (TEM) was used to visualize the morphology of exosomes, with sample preparation for TEM adhering to the negative staining protocol.

## 2.4. Cellular uptake of PKH-26-labeled MSC-Exos

## A PKH-26 labeling kit was employed to label MSC-Exos following the manufacturer’s instructions. The MSC-Exos samples were incubated with 100μM PKH-26 dye solution at 37 °C for 10 minutes in the absence of light. The staining process was halted by adding 10 mL of PBS. Subsequently, PKH-26-labeled MSC-Exos were isolated via differential centrifugation and resuspended in 100 μL of PBS. H9c2 cells were then cultured with the labeled MSC-Exos in a confocal dish for 24 hours. After removing the culture supernatant and washing the cells with PBS, they were stained with 4',6-diamidino-2-phenylindole (DAPI) for 5 minutes. The uptake of PKH-26-labeled MSC-Exos by the cells was visualized using confocal microscopy.

## 2.5. Swelling and degradation test of hydrogels

## The swelling capacity of the hydrogels was determined through a gravimetric method. Initially, the dried hydrogel samples were weighed to record their mass as W_0_. These samples were then placed in PBS at 37°C to allow them to swell. At predetermined intervals, the hydrogels were removed from the PBS, gently blotted with filter paper to eliminate any excess water, and reweighed (W_t_). The swelling ratio for each hydrogel was then calculated using the following formula:Swelling ratio（g/g）= (W_t_ -W_0_)/W_0_×100% where W_t_ indicates the weight of the swollen hydrogel at each time point and W_0_ represents the weight of the lyophilized hydrogel.

## The hydrogels (1 mL) were immersed in PBS containing 0.2 U collagenase and incubated at 37°C. At specified intervals, the hydrogels were removed, lyophilized and weighed to determine the degradation rate. The degradation rate of the hydrogels was calculated using the following equation: degradation rate (%) = (W_0_-W_t_ / W_0_ )×100%, where W_0_ represents the initial mass of the hydrogel and W_t_ is the mass of the hydrogel at the predetermined time points.

## 2.6. Material release test for hydrogels *in vitro*

2.6.1. Metformin

## The *in vitro* release of metformin from hydrogels was studied by immersing the metformin-loaded hydrogels in 3 mL of PBS with varying pH levels (7.4 and 6.8). The samples were agitated at 150 rpm in a shaking bath set to 37 °C. At specified intervals, 1 mL of the PBS release medium was removed and replaced with an equal volume of fresh PBS. The released metformin was then measured using an ultraviolet spectrophotometer (UV-2550, Shimazu, Japan) at 233 nm.

2.6.2. Exosomes

## To prepare the Exo-hydrogel for *in vitro* exosome release testing, 100 μL of hydrogel precursor with PKH26-labeled MSC-Exos was solidified in a 6 mm diameter cylindrical mold at 37°C for 1 hour. The hydrogel was transferred to a 48-well plate and immersed in 1 mL of PBS buffer (pH 7.4 and 6.8) or PBS with 50 U/mL hyaluronidase. Incubation occurred at 37°C in the dark with agitation at 60 rpm. Daily, 100 μL of release buffer was sampled, and its fluorescence intensity at 520 nm (excitation at 480 nm) was measured. An equal volume of fresh medium was replenished. The cumulative release ratio of exosomes was calculated by comparing the photoluminescence intensity of the release solution to the total exosomes in PBS. Each group was tested in triplicate.

## 2.7. *In vivo* tracking of EXOs

## DiR-labeled exosomes were encapsulated within hydrogels comprising OHA/Col-CDH/MWCNT-0.2 and delivered via intramyocardial injection at two predetermined sites within the border zone of infarcted myocardium following LAD ligation. Their distribution and retention were systematically evaluated using *in vivo* small animal imaging at designated time points, specifically on days 1, 3, 5, 10, and 14.

## 2.8. Biosafety Validation

2.8.1. In vitro cell viability

## Cell viability and proliferation of rat embryonic cardiomyocytes (H9c2) and human umbilical vein endothelial cells (HUVEC) were assessed using the Cell Counting Kit-8 (CCK-8) assay. Initially, H9c2 and HUVEC cells were seeded into 96-well plates and cultured at 37°C with 5% CO2 for 24 hours. Following this, the medium was removed, and 100 μL of PBS, hydrogel extract, metformin or exosomes was added to each well. The cells were then incubated with the respective medium for 24, 48, and 72 hours. After each incubation period, the medium was replaced with fresh medium containing 10% CCK-8 and incubated for an additional 2 hours. Absorbance was measured at 450 nm using a microplate reader to determine cell viability. The absorbance of control cells was set as 100%, and relative cell viability was calculated accordingly. Cell viability(%)=AbS_extract/_AbS_control_. The AbS_control_ and AbS_extract_ are the absorbance of cells cultured in DMEM and drugs, respectively.

2.8.2. Calcein-AM (live)/PI (dead) staining

## Cell viability was qualitatively assessed using Calcein-AM (live)/PI (dead) staining. H9c2 and HUVEC cells were seeded in well plates and incubated at 37 °C with 5% CO2 for 24 hours. After removing the medium, 500 μL of PBS, hydrogel extract, metformin or exosomes was added. Following incubation periods of 24, 48, and 72 hours, the cell culture plates were washed with PBS and stained with calcein and propidium iodide solution for 15 minutes at room temperature. Stained cells were then examined under a fluorescence microscope (Olympus, Japan).

2.8.3. Hemolysis experiment

## Rat blood was collected in an anticoagulation tube containing EDTA-Na2, centrifuged at 1000 g for 10 min, the supernatant was removed, and resuspended by adding PBS (pH=7.4), and repeated centrifugation 3 times to remove the collagen fibers. After resuspension of erythrocyte suspensions by PBS, 100 μL was taken and added to EP tubes, followed by 900 deionized water for positive control group, and 900 μL of PBS for negative control group, 900 μL of hydrogel extract was added to the sample group. After mixing, the EP was incubated at 37 ℃ for 120 min, centrifuged at 1000 g for 10 min to precipitate the erythrocytes, and the photographs of EP tubes were collected. Each well was aspirated 200mL of supernatant in a 96-well plate, and the absorbance value at 540nm was detected by an enzyme marker, and the hemolysis of each group was calculated according to the following formula: Hemolysis (%) = [(OD_sample_–OD_negative control_)/(OD_positive control_–OD_negative control_)] × 100%.

2.8.4. Histopathological examination

Upon completion of the experiment, the rats were euthanized, and tissues from various organs (lung, liver, spleen, and kidney) were harvested for histopathological examination. Hematoxylin and eosin (HE) staining was performed on these tissue samples to assess the *in vivo* biosafety of the hydrogel.

## 2.9. Intracellular ROS was detected by dichlorodihydrofluorescein diacetate (DCFH-DA)

The DCFH-DA stock solution was diluted to a final concentration of 10μM using serum-free culture medium. The cell culture medium was carefully aspirated, and the cells were washed with an appropriate amount of serum-free culture medium. The diluted DCFH-DA working solution was added to the cell culture, ensuring complete coverage of the cells. The cells were incubated at 37°C with 5% CO₂ for 30 minutes to 1 hour in the dark. After incubation, the DCFH-DA working solution was aspirated, and the cells were washed 2-3 times with serum-free culture medium or PBS to remove any unbound dye. A fluorescence microscope was set up with appropriate excitation (typically 485 nm) and emission (typically 530 nm) filter sets. The cells were then observed under the confocal laser scanning microscopy (CLSM).

H9c2 cells were stimulated as per previously described protocols, and DCFH-DA was subsequently added to the cell suspension. Following stimulation, the cells were harvested by centrifugation in a temperature-controlled water bath, resuspended, and transferred to flow cytometry tubes. Intracellular ROS levels were quantified using flow cytometry.

## 2.10. Apoptosis detection

2.10.1. Annexin V-FITC/PI

## According to the instructions, dye was added to each group, and cells were collected after the designated intervention period. Following centrifugation, the cells were resuspended in binding buffer and stained with Annexin V-FITC and PI solutions. The samples were then analyzed using flow cytometry (Agilent, China).

2.10.2. TUENL

The cells were fixed with 4% paraformaldehyde solution for 10-20 minutes and then washed with PBS. The cell membrane was permeabilized using a 0.2% Triton X-100 solution. The TUNEL reaction mixture was prepared according to the kit instructions. An appropriate amount of the TUNEL reaction mixture was added to cover the cells, and the cells were incubated at 37°C in the dark for 1 hour. Finally, the cells were observed using a fluorescence microscope.

## 2.11. Calcium-Transient Studies

Sprague–Dawley rats aged 1–3 days were used in this study. Neonatal rat cardiomyocytes (NRCMs) were isolated and seeded onto conductive hydrogels.  The cells were then cultured for 7 days at 37 °C in a humidified environment containing 5% CO2 . To assess intracellular Ca2+ transients in CMs seeded on the hydrogel, all samples cultured for 7 days were stained with a calcium indicator (50 μg Fluo-4 AM) at 37 °C for 45 min, followed by observation of intracellular calcium fluorescence under a (CLSM).

## 2.12. Cell immunofluorescence

After treating the NRCMs according to their respective experimental groups, NRCMs were fixed with 4% paraformaldehyde for 1 hour to preserve cellular structures. The cells were then permeabilized with 0.3% Triton X-100 in PBS for 15 minutes to facilitate antibody access. To block nonspecific protein interactions, the samples were incubated with 2% bovine serum albumin (BSA) at 4°C for 2 hours. Following this step, primary antibody incubation was performed overnight at 4°C to ensure specific antigen-antibody binding. The next day, the samples were rinsed three times with PBS and subsequently incubated with a secondary antibody for 2 hours at room temperature on a shaker to enhance signal detection. Lastly, the nuclei of the NRCMs were counterstained with DAPI for 10 minutes, and the samples were analyzed using CLSM.

## 2.13. Cellular MMP Assay: JC-1 Assay

After washing the cells with PBS, 1 mL of culture medium was added to maintain a suitable environment. Subsequently, 1 mL of JC-1 staining working solution (Beyotime Biotechnology) was carefully introduced and thoroughly mixed to ensure uniform staining. The cells were incubated at 37°C in a humidified incubator for 20 minutes to facilitate dye uptake. Following incubation, the supernatant was carefully removed, and the cells were rinsed twice with JC-1 staining buffer to eliminate excess dye. Finally, 2 mL of fresh culture medium was added to the cells, and their fluorescence was observed and imaged using a fluorescence microscope.

## 2.14. Mitochondrial Morphology Assay

CMs from all experimental groups were first incubated in 2.5% glutaraldehyde at room temperature for 5 minutes under light-protected conditions to preserve cellular structures. The cells were then subjected to centrifugation at 2500 rpm for 2 minutes to form a pellet. Subsequently, the glutaraldehyde was replaced with fresh electron microscopy fixative to enhance preservation, and the CMs were gently resuspended in the fixative. Finally, the samples were sectioned and stained to prepare them for detailed examination via transmission electron microscopy (TEM).

## 2.15. Scratch test

## The migratory capacity of HUVEC was evaluated using a scratch test. HUVEC (1X 10^6^)were seeded into a 6-well plate and cultured in a standard medium containing 10% fetal bovine serum (FBS). Upon reaching over 90% confluence, a scratch was created in the cell monolayers with a sterile 200 μL micropipette tip. The cells were then incubated for 24 hours in media containing 1% FBS with various treatments: hydrogel, hydrogel with metformin (400 μmol/mL), hydrogel with exosomes (20 μg/mL), and hydrogel with both metformin (400 μmol/mL) and exosomes (20 μg/mL), the cells except those in the normal group were then treated with H/R. Images of three fields of view were captured at 0, 12, 24, and 36 hours using an inverted microscope. The migration distance was analyzed and quantified using Image J software.

## 2.16. Tube Formation Assay

## To evaluate the proangiogenic effects of exosomes, a tube formation assay was conducted. The BD Matrigel matrix was kept on ice overnight prior to the test. On the day of the experiment, 200 μL of Matrigel was added to each well of a 48-well plate and allowed to polymerize for 1 hour at 37 °C with 5% CO_2_. For the assessment of capillary-like structure formation by HUVEC, growth factor-reduced Matrigel (BD Biosciences) was mixed 1:1 with cold DMEM on ice. HUVEC were cultured for 24 hours in media containing 10% FBS with different treatments: hydrogel, hydrogel with metformin (400 μmol/mL), hydrogel with exosomes (20 μg/mL), and hydrogel with both metformin (400 μmol/mL) and exosomes (20 μg/mL), the cells except those in the normal group were then treated with H/R. Subsequently, 3X10^4^ cells per well were seeded onto the Matrigel-coated plates. After 12 hours of incubation at 37°C with 5% CO_2_, images were captured using an Olympus bright-field microscope. The proangiogenic activity of HUVEC was quantified using Image J software by measuring the number of junctions, branches, and nodes in three random fields per well.

## 2.17. Transwell assay

## HUVECs were cultured for 24 hours in media containing 10% FBS and subjected to various treatments: hydrogel alone, hydrogel with metformin (400 μmol/mL), hydrogel with exosomes (20 μg/mL), and hydrogel with both metformin (400 μmol/mL) and exosomes (20 μg/mL). The cells except those in the normal group were then treated with H/R. The cells were then prepared in a serum-free DMEM medium at a concentration of 5X10^4^ cells/mL and added to the upper chamber of a transwell insert. The lower chamber was filled with culture medium containing the corresponding treatment suspensions. After a24-hour incubation period at 37°C, cells were fixed with paraformaldehyde, stained with crystal violet, and subsequently analyzed under a microscope to evaluate their migratory ability. Cell migration was quantified using Image J software.

## 2.18. Transcriptome sample collection and preparation

H9c2 cells (2.0×10⁷) were cultured in dishes and, upon reaching approximately 80% confluence, were separated into three experimental groups: Control, H/R, and Met+Exo+Gel. The respective treatments were then applied to each group. Total RNA was extracted using TRIzol reagent (Ambion) following the protocol provided by the manufacturer. The integrity of the RNA samples was assessed using the RNA Nano 6000 Assay Kit and the Bioanalyzer 2100 system (Agilent Technologies). Subsequently, the total RNA was used to generate an RNA library through amplification via polymerase chain reaction. The quality of the RNA library was verified with the Agilent 2100 Bioanalyzer. Once qualified, the libraries were pooled based on their effective concentrations and the desired target data output for sequencing on an Illumina platform.

**2.19. Transcriptome data analysis**

To ensure data quality and reliability, the original data underwent filtering (quality control). Subsequently, differential gene expression analysis was conducted using DESeq2 software (version 1.20.0) on two comparative groups (n = 3). The Benjamini and Hochberg method was employed to adjust p-values, thereby controlling the false discovery rate. Genes with a p-value < 0.05 were deemed differentially expressed according to DESeq2. Further analysis included Gene Ontology (GO), Kyoto Encyclopedia of Genes and Genomes (KEGG) enrichment, and Gene Set Enrichment Analysis (GSEA) for the differentially expressed genes.

**2.20. Echocardiography studies**

An ultrasound imaging system was employed to assess left ventricular (LV) function in all experimental groups. Four weeks after the injection, echocardiographic recordings were obtained while the rats were anesthetized using isoflurane. Transthoracic two-dimensional guided M-mode imaging was utilized to obtain a short-axis view at the level of the papillary muscles. Parameters including ejection fraction (EF), fractional shortening (FS), LV internal dimensions at end-systole (LVIDs) and end-diastole (LVIDd), as well as end-systolic volume (ESV) and end-diastolic volume (EDV), were measured. Each value represented the average of three consecutive cardiac cycles for improved accuracy.

**2.21. Programmed electrical stimulation**

Four weeks post-hydrogel injection, arrhythmia inducibility was evaluated using a clinically standardized programmed electrical stimulation (PES) protocol. This protocol consisted of a series of stimuli applied under spontaneous rhythm conditions, including burst pacing with a cycle length of 120 ms, followed by single (70 ms), double (60 ms), and triple (50 ms) additional stimuli. The susceptibility to arrhythmias was quantified through the inducibility quotient, defined as follows. 0: Absence of premature ventricular contractions (PVCs) or ventricular tachycardia (VT); **1**: Non-sustained PVCs or VT (≤15 beats) elicited by three extra stimuli; **2**: Sustained PVCs or VT (>15 beats) induced by three extra stimuli; **3**: Non-sustained PVCs or VT induced by two extra stimuli; **4**: Sustained PVCs or VT elicited by two extra stimuli; **5**: Non-sustained PVCs or VT triggered by a single extra stimulus; **6**: Sustained PVCs or VT induced by a single extra stimulus; **7**: Sustained or non-sustained PVCs or VT occurring following a train of eight stimuli; 8: Asystole observed after the termination of pacing; Higher inducibility quotient values indicated an increased risk of arrhythmia.

## 2.22. Histological analysis

## The heart tissues were preserved in 4% paraformaldehyde, embedded in paraffin, and sectioned into 4-μm-thick slices. These sections underwent staining with hematoxylin and eosin (H&E), Masson's trichrome following the protocols of manufacturer. For immunofluorescence analysis, the tissue sections were first incubated overnight at 4°C with primary antibodies, including terminal deoxynucleotidyl transferase dUTP nick end labeling (TUNEL), α-actin (11313-2-AP), cTnT (21652-1-AP), Connexin 43 (CX43) (26980-1-AP), α-smooth muscle actin (α-SMA) (14395-1-AP), CD31 (28083-1-AP), vWF (27186-1-AP), Ki67 (27309-1-AP), and vascular endothelial growth factor (VEGF) (19003-1-AP) from Proteintech. Following this, they were incubated for 1 hour at 37°C in the dark with secondary antibodies, specifically Alexa Fluor antibodies from Abcam, including goat anti-rabbit 488 (ab150077), goat anti-mouse 488 (ab150117), and goat anti-rabbit 555 (ab150078). Subsequently, the nuclei were stained with DAPI. Fluorescence microscopy was used to observe and capture images.

**Table S1.** Detailed compositions of the four hydrogels.

| Sample | OHA^a)^  (w/v%) | Col-CDH^b)^  (w/v%) | MWCNT^c)^  (w/v%) | MSC-Exos^d)^  （μg/mL） | Metformin  (mg/mL) |
| --- | --- | --- | --- | --- | --- |
| OHA/Col-CDH | 10 | 10 | 0 | 100 | 4 |
| OHA/Col-CDH/  MWCNT-0.1 | 10 | 10 | 0.1 | 100 | 4 |
| OHA/Col-CDH/  MWCNT-0.2 | 10 | 10 | 0.2 | 100 | 4 |
| OHA/Col-CDH/  MWCNT-0.5 | 10 | 10 | 0.5 | 100 | 4 |

^a)^(oxidized hyaluronic acid); ^b)^(carbohydrazide-modified collagen); ^c)^(multi-walled carbon nanotubes); ^d)^(mesenchymal stem cell exosomes).

3. Results


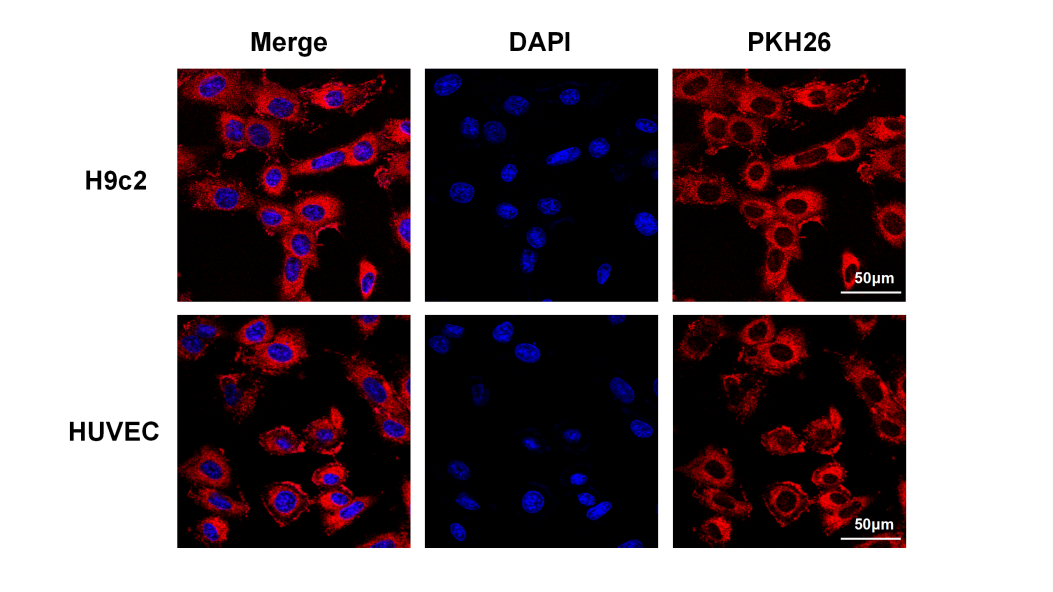


**Figure S1.** Microscopic view of exosomes labelled by PKH26 being taken up by H9c2 and HUVEC. Scale bars: 50µm.


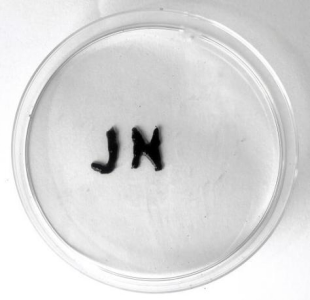


**Figure S2.** Demonstration of the injectability of the hydrogel.


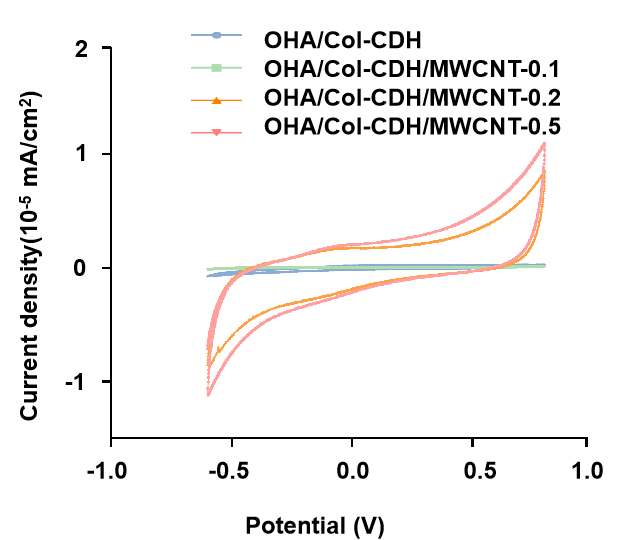


**Figure S3.** Cyclic voltammetry curves for various hydrogels.

**Figure S4.** Quantitative analysis of the transwell experiment. Statistical data by means ± SD (n=5). One-way ANOVA was used to compare multiple groups. ** *p* < 0.01, *** *p* < 0.001, **** *p* < 0.0001.

**Enriched GO terms** **(H/R vs Control)**


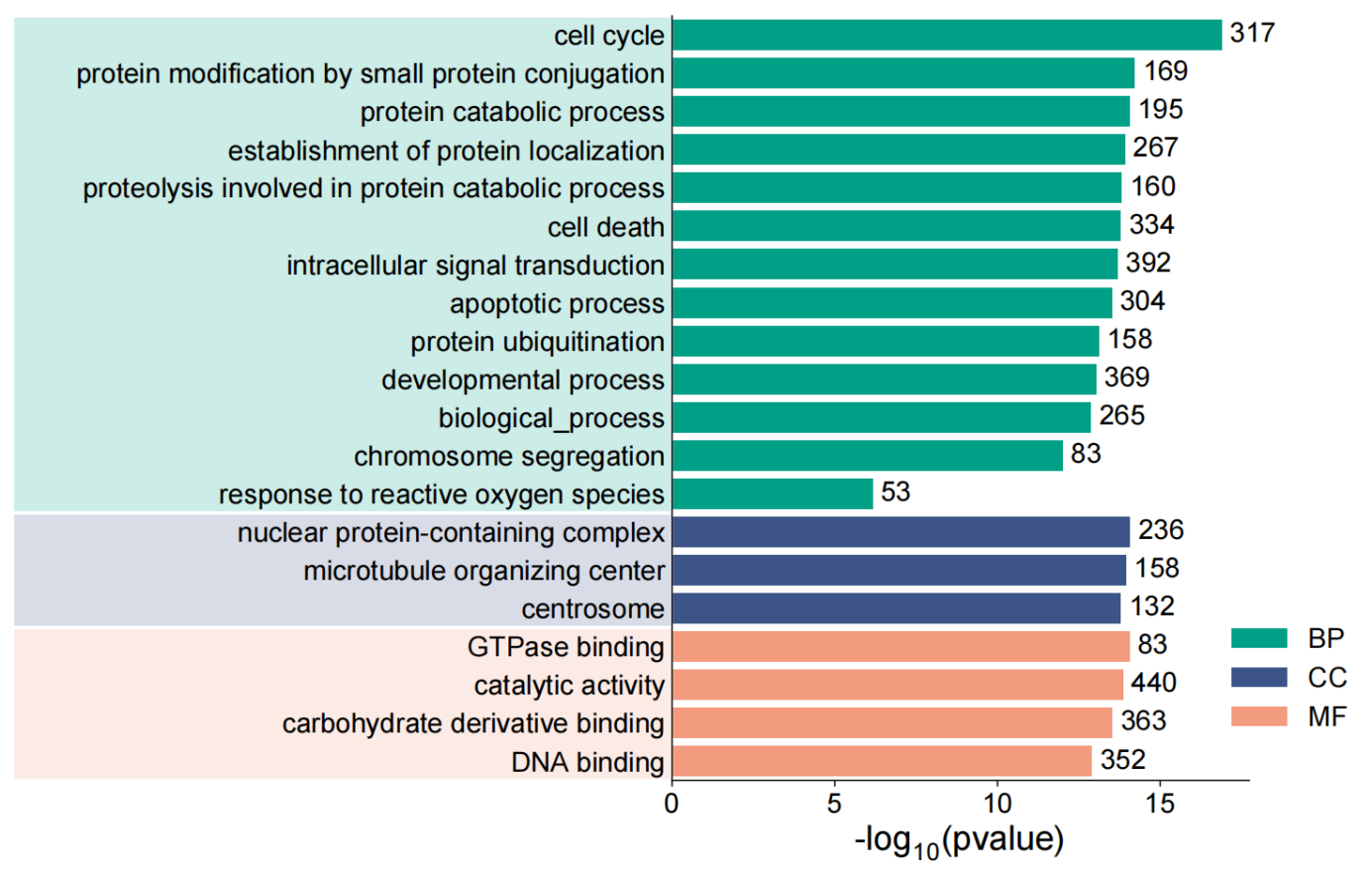


**Figure S5.** GO analysis of DEGs among H/R versus Control groups.

**KEGG Pathway Enrichment TPO 20 (H/R vs Control)**


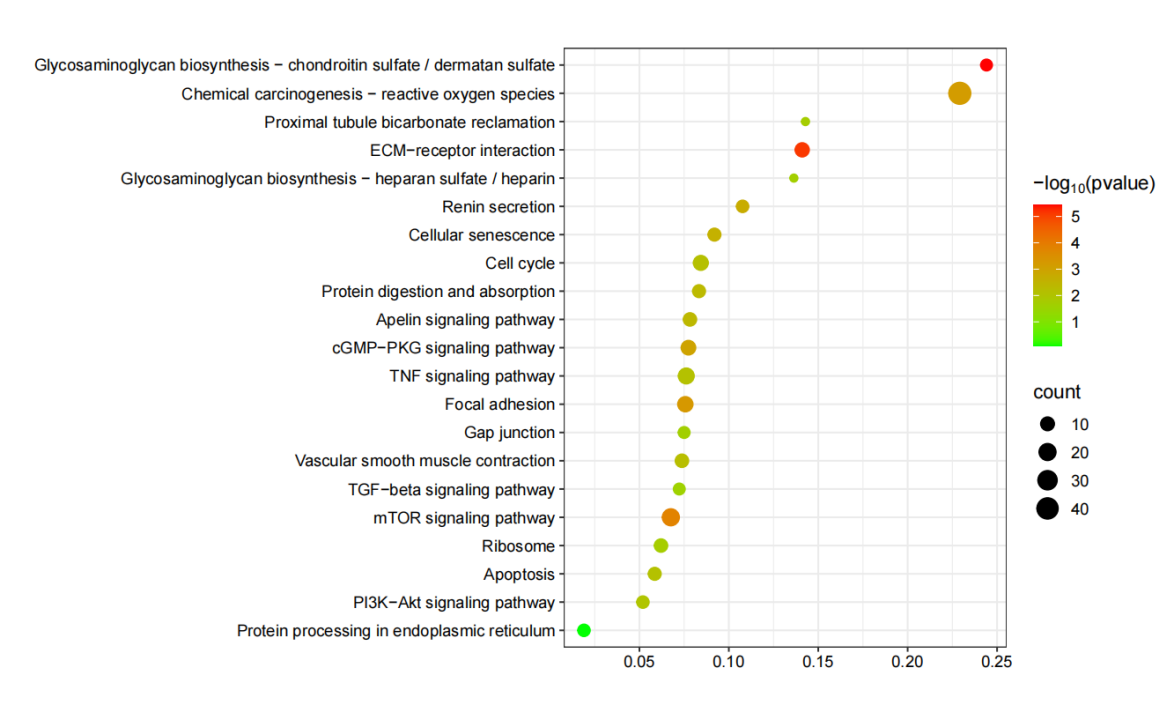


**Figure S6.** KEGG pathway enrichment analysis of DEGs among H/R versus Control groups.


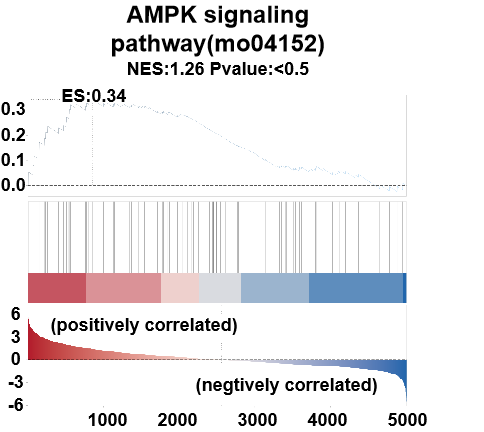


**Figure S7.** GSEA of AMPK signaling pathway among Met+Exo+Gel versus H/R groups.


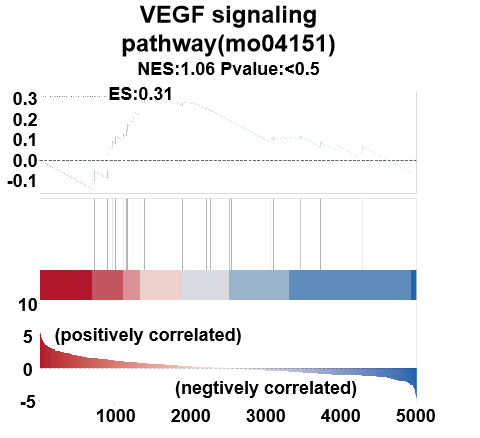


**Figure S8.** GSEA of VEGF signaling pathway among Met+Exo+Gel versus H/R groups.


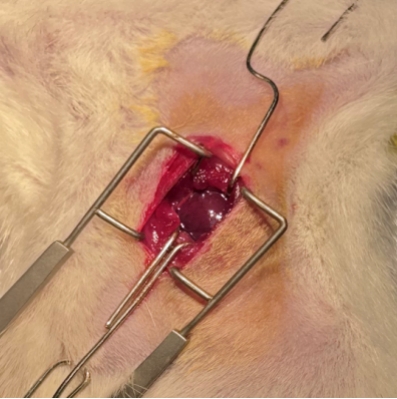


**Figure S9.** MIRI model operation real picture.


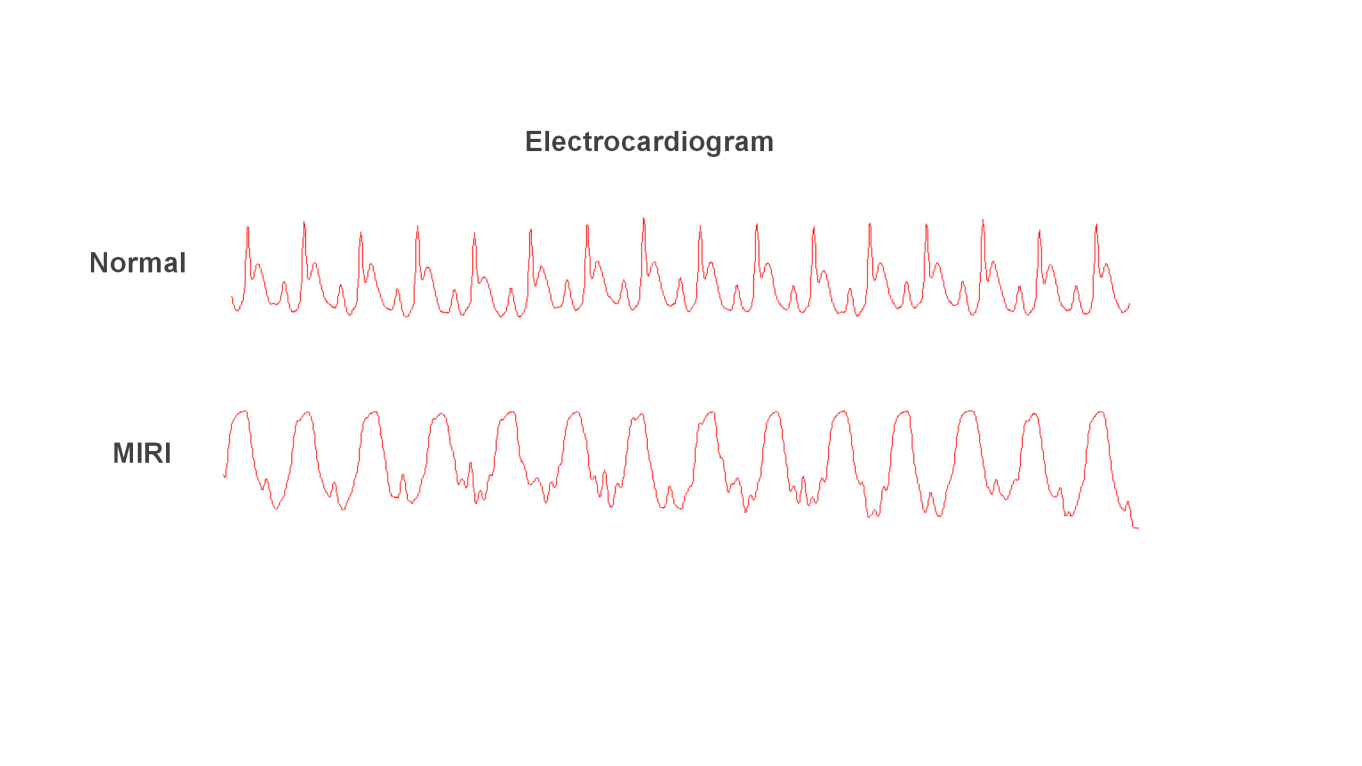


**Figure S10.** The MIRI model was confirmed by electrocardiogram.

**Figure S11.** LVIDd were determined by echocardiography after treatment. Statistical data are expressed as mean ± SD (n = 5). One-way ANOVA was used to compare multiple groups. * *p* < 0.05, ** *p* < 0.01, *** *p* < 0.001,**** *p* < 0.0001.

**Figure S12.** LVIDs were determined by echocardiography after treatment. Statistical data are expressed as mean ± SD (n = 5). One-way ANOVA was used to compare multiple groups. * *p* < 0.05, ** *p* < 0.01, *** *p* < 0.001, **** *p* < 0.0001.


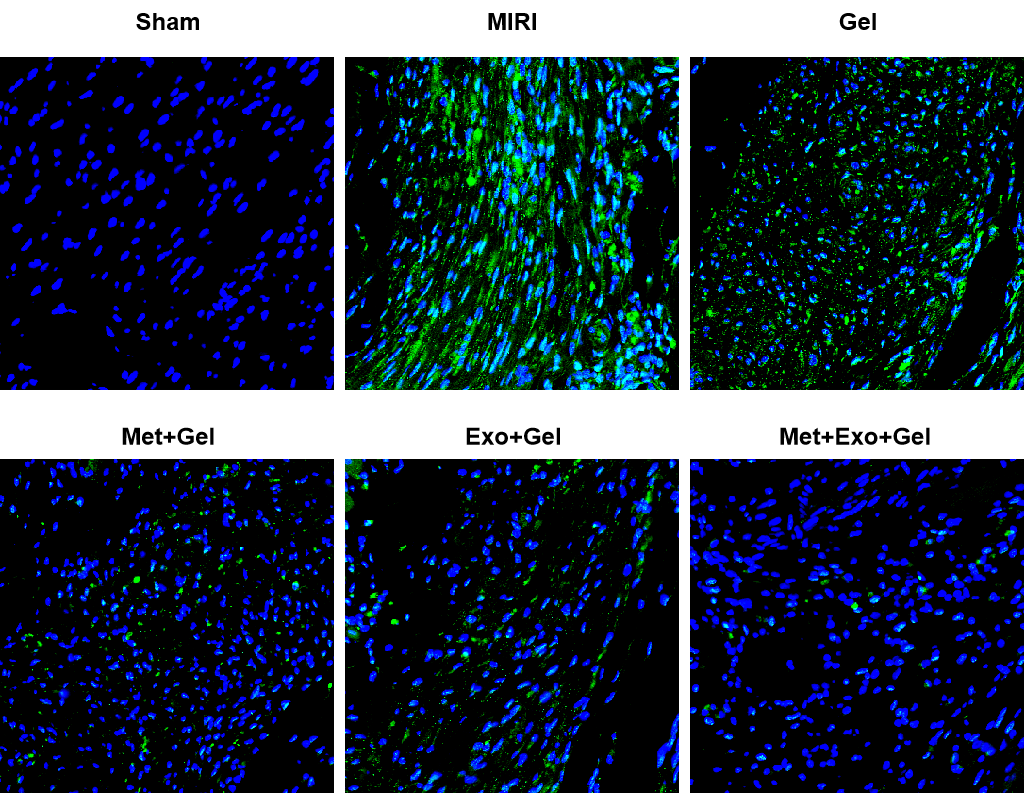


**Figure S13.** ROS expression in infarcted area after different treatments *in vivo*.

**Figure S14.** Quantitative analysis of ROS relative intensity fluorescence. Statistical data by means ± SD (n = 5). One-way ANOVA was used to compare multiple groups. * *p* < 0.05, ** *p* < 0.01, *** *p* < 0.001, **** *p* < 0.0001.


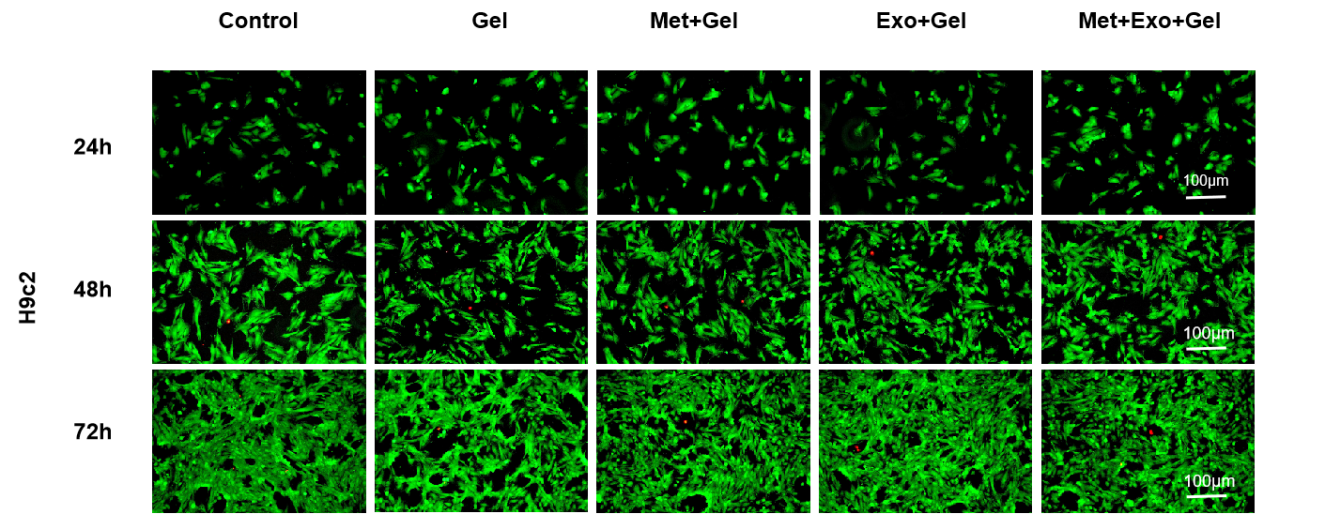


**Figure S15.** Calcein-AM/PI staining of H9c2 cells treated with hydrogel treatment system. Scale bars: 100 µm.


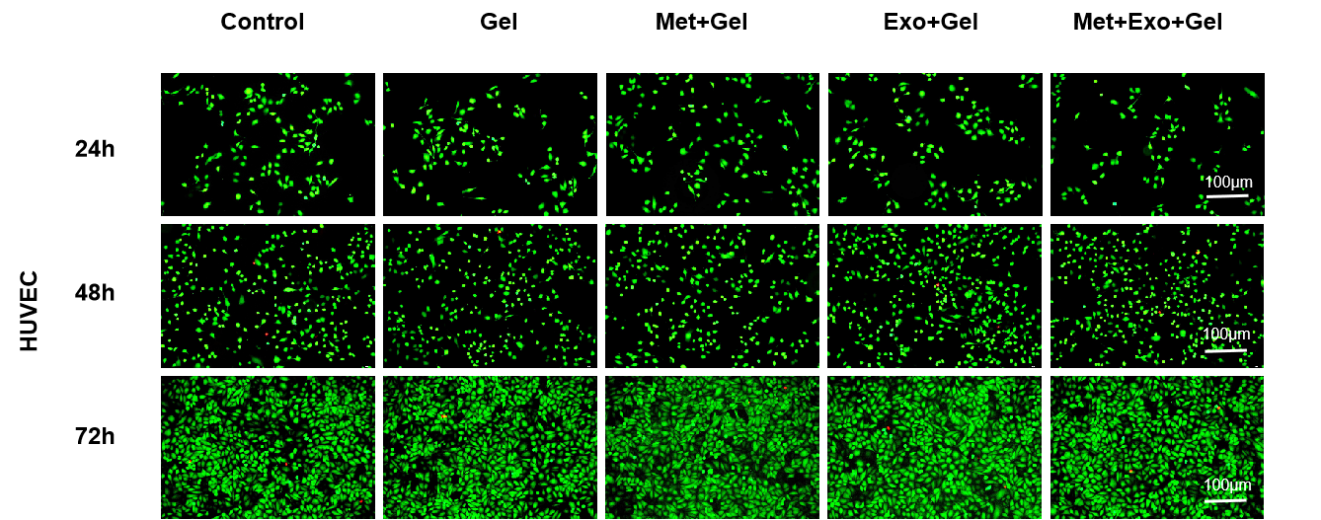


**Figure S16.** Calcein-AM/PI staining of HUVECs treated with hydrogel treatment system. Scale bars: 100 µm.

**Figure S17.** Cell viability of H9c2 treated with hydrogel treatment system. Statistical data by means ± SD (n = 3).

**Figure S18.** Cell viability of HUVEC treated with hydrogel treatment system. Statistical data by means ± SD (n = 3).

**Figure S19.** Demonstration of the hemocompatibility of the hydrogel treatment system. Statistical data by means ± SD (n = 3).


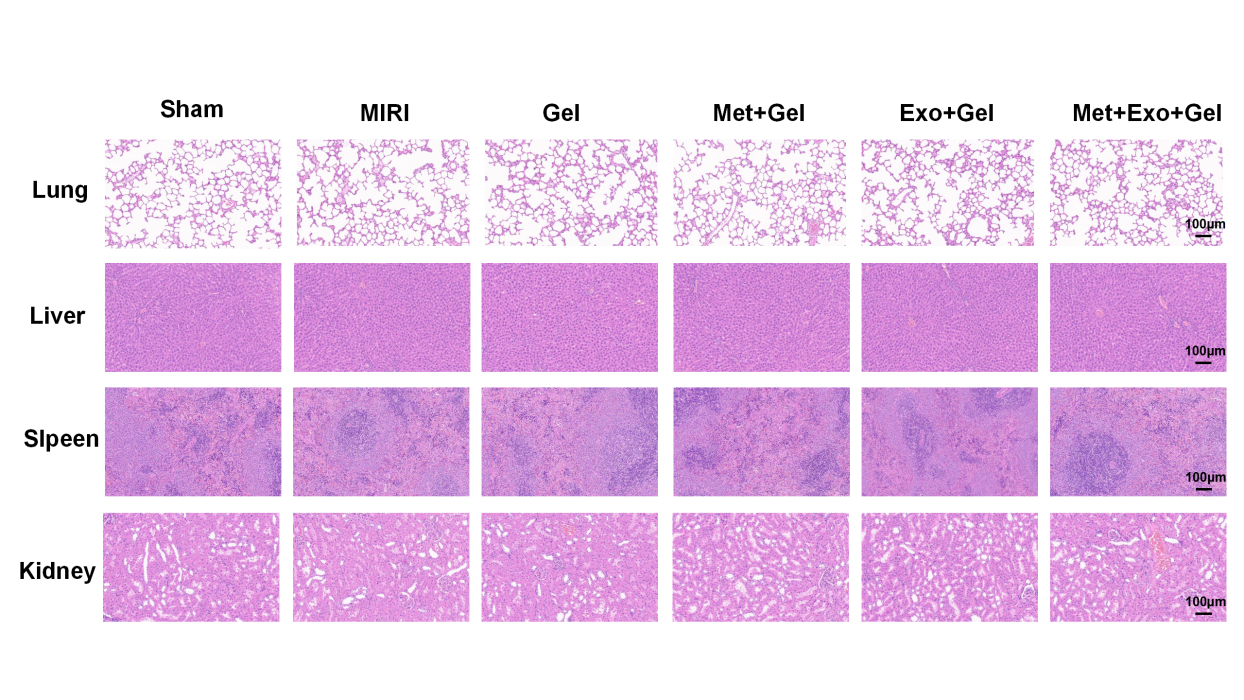


**Figure S20.** H&E analysis of major organs after treatment. Scale bars: 100 µm.

**References**

[1] Y. Huang, B. He, L. Wang, B. Yuan, H. Shu, F. Zhang,L. Sun, *Stem Cell Res Ther.* **2020**, *11*, 496.
